# Impacts of optimal control strategies on the HBV and COVID-19 co-epidemic spreading dynamics

**DOI:** 10.1038/s41598-024-55111-8

**Published:** 2024-03-04

**Authors:** Shewafera Wondimagegnhu Teklu

**Affiliations:** Department of Mathematics, Natural Science, Debre Berhan University, Debre Berhan, Ethiopia

**Keywords:** HBV, COVID-19, Co-epidemic, Vaccination, Protection, Optimal control measures, Computational biology and bioinformatics, Health occupations, Mathematics and computing

## Abstract

Different cross-sectional and clinical research studies investigated that chronic HBV infected individuals’ co-epidemic with COVID-19 infection will have more complicated liver infection than HBV infected individuals in the absence of COVID-19 infection. The main objective of this study is to investigate the optimal impacts of four time dependent control strategies on the HBV and COVID-19 co-epidemic transmission using compartmental modeling approach. The qualitative analyses of the model investigated the model solutions non-negativity and boundedness, calculated all the models effective reproduction numbers by applying the next generation operator approach, computed all the models disease-free equilibrium point (s) and endemic equilibrium point (s) and proved their local stability, shown the phenomenon of backward bifurcation by applying the Center Manifold criteria. By applied the Pontryagin’s Maximum principle, the study re-formulated and analyzed the co-epidemic model optimal control problem by incorporating four time dependent controlling variables. The study also carried out numerical simulations to verify the model qualitative results and to investigate the optimal impacts of the proposed optimal control strategies. The main finding of the study reveals that implementation of protections, COVID-19 vaccine, and treatment strategies simultaneously is the most effective optimal control strategy to tackle the HBV and COVID-19 co-epidemic spreading in the community.

## Introduction

Pathogenic microbial agents such as funguses, viruses, bacteria and parasites have been the most causative agents of infectious diseases. HBV and COVID-19 co-epidemic is an infectious disease caused by two virus pathogens and has been affecting the life of million individuals in various nations throughout the world^1–3^.

Different studies reveal that HBV is one of the microbial pathogenic viruses that commonly influencing the work of individuals’ liver and has been a cause for death of millions of individuals with its chronic stages liver cirrhosis and cancer^3,4^. HBV spreads in the population through direct and indirect transmission like with blood contact or fluids of infectious people or during child birth^2,5^.

The pandemic of COVID-19 in 2020 has yet to be fundamentally contained and it puts a long pause button on the lives of people around the globe^6^. The acute respiratory infectious disease COVID-19 was discovered in China and has been declared as a world pandemic contagious disease^7–14^. Due to its very high spreading rate, on March 11, 2020, WHO explained it as a worldwide pandemic infectious disease^10,15,16^. Individuals can acquire COVID-19 infection directly from other individuals by touching contaminated objects and indirectly by air droplets inhalation from other individuals sneezing or/and coughing^17–19^. It negatively affects the world nations’ health policies, economies and population densities^20,21^. The protection and control measures explained by WHO are quarantine, applying face masks, hand washing, isolation, vaccination, maintaining social distance and treatment strategies^17,21,22^.

Different researchers throughout nations in the world have studied about the co-interaction of COVID-19 with various infectious diseases like tuberculosis (TB), HIV/AIDS, HBV and cholera^23–28^. The studies carried out in references^4–23,29–45^, formulated and analyzed the spreading dynamics of infectious diseases using compartmental integer order modeling approach, studies carried out in references^3,28^ formulated and examined the spreading dynamics of infectious diseases using stochastic modeling approach, and studies carried out in references^24,26,36,46–50^ are formulated and analyzed using fractional order modeling approach.

Investigating and predicting the spreading rates of infectious diseases using compartmental modeling approaches has fundamental effects to tackle the spreading problem in the community^8^. To construct and examined the HBV and COVID-19 co-epidemic compartmental integer order model we have reviewed the following published research literatures by other scholars in different countries throughout the world: Baba et al.^42^, formulated a novel COVID-19 mathematical model to investigate the imposition of lockdown during its pandemic. Their main objective is to study and investigate the imposition of lock-down on the dynamics of COVID-19 in Nigeria. Ibrahim et al.^43^ constructed and analyzed a COVID-19 model by applying real data from Thailand. From their model analysis results to tackle the problem they suggested that public health policymakers should prioritize increasing the intervention strategies such as vaccination coverage, enhancing testing and tracing capacities, enforcing social distancing and mask wearing measures, and monitoring the emergence and spread of new variants. Li and Guo^6^ formulated and examined a mutated COVID-19 (delta strain) mathematical model and the corresponding optimal control problem with imperfect vaccination. Their findings suggested that the optimal control strategy is to dynamically adjust the three control measures to achieve the lowest number of infections at the lowest cost. Guo and Li^14^ constructed and analyzed novel coronavirus pneumonia (COVID-19) in China. Their analysis result proves that theoretically the Chinese government’s epidemic prevention strategies are effective to control the spread of COVID-19 infection. Tchoumi et al.^8^ examined the optimal control measures on the COVID-19 and malaria co-epidemic by constructing its compartmental model. The finding of their study shows that the combined implementation of protection strategies is the best control measure to minimize the co-epidemic in the population. Teklu and Koya^31^ developed pneumonia and HIV/AIDS co-infection and examined the impacts of treatment and vaccination controlling measures to tackle the spreading problem. Hezam et al.^23^, investigated the impacts of prevention measures on the co-infection of cholera and COVID-19 in Yemen by applying compartmental modeling method. Anwar et al.^22^, examined the impact of isolation controlling strategy for COVID-19 spreading in the population by using qualitative and numerical analysis of their compartmental model. Ahmed et al.^24^ and Ringa et al.^11^ studied HIV/AIDS and COVID-19 co-infection using fractional order and integer order modeling approach respectively. From their findings one can observe that protection strategies are most efficient strategies to tackle the co-infection spreading in the population. Din et al.^3^ and Omame et al.^51^ constructed a compartmental model on the spreading of HBV and COVID-19 co-epidemic through the community using mathematical modeling approach and investigated the ways how to control the spreading of the co-infection. Din et al.^28^ constructed HBV and COVID-19 co-epidemic stochastic model with limitation of resources. The study investigates the fluctuation of the stochastic model HBV and COVID-19 co-epidemic disease-free equilibrium point using Lyapunov function method and the numerical results justifies the qualitative results. Teklu^2^ formulated and examined the HBV and COVID-19 co-infection compartmental model to investigate the effects of some prevention and controlling strategies without applying optimal control theory. Li et al.^52^ constructed the generalized COVID-19 deterministic model to investigate the epidemiological characteristics. Studies carried out in references^2,3,26,28,51^ investigated the co-existence spreading of HBV and COVID-19.

According to various mathematical modeling research studies of infectious diseases especially on HBV and COVID-19 infection reviewed in our research process, none of them considered to study the impacts of our proposed four time dependent control strategies on the HBV and COVID-19 co-epidemic model that incorporate acute and chronic HBV infection stages, protection for both infections, and COVID-19 vaccination measures and these makes our model novel as compared with previously HBV and COVID-19 co-infection models formulated by other scholars. As a result of these scientific gaps the author motivated to achieve the main objective of this paper that is to investigate the impact of vaccination, protection and treatment strategies for the prediction and tackling of the HBV and COVID-19 co-epidemic spreading in the community by formulating a novel HBV and COVID-19 co-epidemic model.

The rest part of this paper is organized in different sections as: section “Descriptions and model construction” discussed procedures of the models formulations with its qualitative analyses carried out in section “Qualitative analysis of the dynamical system”, the optimal control problem in section “The optimal control problem and its qualitative analysis”, the numerical analysis in section “Numerical simulations”, and the conclusion of the whole study in section “Conclusion”.

## Descriptions and model construction

In this sub-section, we need to formulate the integer order model on HBV and COVID-19 co-epidemic spreading dynamics in the community by partitioning the human host population $N\left( t \right)$ into eleven distinct mutually exclusive groups as: healthy people who are susceptible to either of COVID-19 or HBV single infection $\left( {S\left( t \right)} \right)$, individuals who can be protected against COVID-19 denoted by ($C_{P} \left( t \right))$, individuals who take protection against HBV denoted by ($H_{P} \left( t \right))$, individuals who take vaccine against COVID-19 denoted by ($C_{V} \left( t \right))$, individuals who are COVID-19 infectious denoted by $\left( {C_{I} \left( t \right)} \right)$, acute HBV infected people ($H_{A} \left( t \right)),$ chronic HBV infected people $(H_{C} \left( t \right))$, people who are co-epidemic by acute HBV and COVID-19 denoted by ($I_{AC} \left( t \right))$, individuals who are co-epidemic by chronic HBV and COVID-19 denoted by ( $I_{CC} \left( t \right))$, individuals who are recovered against COVID-19 single infection denoted by $\left( {C_{R} \left( t \right)} \right)$, and people who are treated from chronic HBV infection $(H_{T} \left( t \right))$ with total population given by

$$ N\left( t \right) = S\left( t \right) + C_{P} \left( t \right) + H_{P} \left( t \right) + C_{V} \left( t \right) + C_{I} \left( t \right) + H_{A} \left( t \right) + H_{C} \left( t \right) + I_{AC} \left( t \right) + I_{CC} \left( t \right) + C_{R} \left( t \right) + H_{T} \left( t \right). $$

Because HBV is a chronic communicable infection, healthy individuals can be infected by HBV at the infection rate illustrated by

1 $$ \lambda_{H} \left( t \right) = \frac{{\beta_{1} }}{N}\left( {H_{A} \left( t \right) + \rho_{1} H_{C} \left( t \right) + \rho_{2} I_{AC} \left( t \right) + \rho_{3} I_{CC} \left( t \right)} \right), $$

where $\rho_{3} \ge \rho_{2} \ge \rho_{1} \ge 1$ are the proposed model parameters that modifies the infectivity of HBV and the parameter ${\upbeta }_{1}$ is the rate of HBV spreading.

Because COVID-19 is an acutely communicable disease, healthy individuals acquires COVID-19 at infection rate illustrated by

2 $$ {\uplambda }_{{\text{C}}} \left( {\text{t}} \right) = {\upbeta }_{2} \left( {C_{I} \left( {\text{t}} \right) + {\upomega }_{1} I_{AC} \left( {\text{t}} \right) + {\upomega }_{2} I_{CC} \left( {\text{t}} \right)} \right),{ } $$

whenever ${\upomega }_{2} \ge {\upomega }_{1} \ge 1$ are the proposed model parameters which modifies the infectivity of COVID-19 and the model parameter ${\upbeta }_{2}$ is the rate of COVID-19 spreading.

Other basic assumptions to construct the proposed HBV and COVID-19 co-epidemic compartmental model: the total human host population recruitment rate be $${\Delta } = b*N$$ where $$b $$ be the human birth rate, $$N$$ be the total number of population, the portions $$k_{1}$$, $$k_{2}$$,$$ k_{3} ,$$ and $$k_{4}$$ with total sum 1 of the recruited individuals ($${\Delta }$$) respectively are individuals go to the susceptible (healthy) group, the COVID-19 protection group, the HBV protection group and the COVID-19 vaccinated group, vaccine against COVID-19 will not be 100% efficient, hence individuals vaccine against COVID-19 will have a probability to be infected with infected with COVID-19 at some portion given by $$ \varepsilon \user2{ }$$ for the serotype that will not be addressed by vaccine whenever $$ 0 \le \varepsilon < 1$$, the number of human host population is variable and homogeneously mixing in each group, treated individuals against HBV do not spread HBV to others, and there is no dual-infection simultaneous spreading.

**Figure 1:**
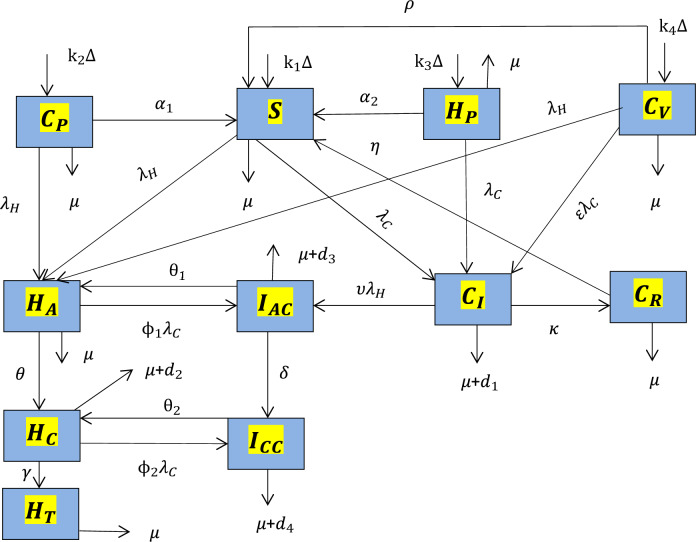
The COVID-19 and HBV co-epidemic individuals flow diagram where the functions λ_*H*_(*t*) and λ_*C*_(*t*) are described in Eqs. (1) and (2) respectively.

Using the assumptions, descriptions in Tables 1 and 2, we construct the HBV and COVID-19 co-epidemic individuals’ flow diagram illustrated with Fig. 1 below.

**Table 1:** Parameter descriptions

Parameters	Descriptions
$\mu$	Natural death rate
${\Delta }$	Recruited individuals
$\alpha_{1}$	The rate at which individuals lose COVID-19 protection
$\alpha_{2}$	The rate at which individuals lose HBV protection
$\varepsilon$	Portion of individuals that do not covered by vaccine
${\uptheta }$	The rate of progression
$\phi_{1}$	The parameter of modification
$\phi_{2}$	The parameter of modification
$d_{1}$	COVID-19 disease death rate
$d_{2}$	Chronic HBV disease death rate
$\kappa$	COVID-19 recovery rate
$\gamma$	Treatment rate of acute HBV infection
$\rho$	Waning rate of COVID-19 vaccination
$\upsilon$	The modification parameter
$\beta_{1}$	HBV spreading rate
$\beta_{2}$	COVID-19 spreading rate
${\text{k}}_{1}$	Portion of recruited individuals go to susceptible group
${\text{k}}_{2}$	COVID-19 protection rate
${\text{k}}_{3}$	HBV protection rate
${\text{k}}_{4}$	COVID-19 rate of vaccine
$\delta$	The progression rate of the co-epidemic
${\uptheta }_{1}$	COVID-19 treatment rate against acute HBV co-epidemic
${\uptheta }_{2}$	COVID-19 treatment rate against chronic HIV co-epidemic
$\eta$	Temporary immunity development rate
$d_{3}$	Death rate for acute HBV and COVID-19 co-epidemic
$d_{4}$	Death rate for chronic HBV and COVID-19 co-epidemic

**Table 2:** Descriptions of the state variables

State variable	Descriptions
$S$	HBV or COVID-19 susceptible group
$C_{P}$	COVID-19 protected individuals
$H_{P}$	HBV protected individuals
$C_{V}$	COVID-19 vaccine group
$C_{I}$	Individuals who are infected with COVID-19
$H_{A}$	Acute HBV mono-infected individuals
$H_{C}$	Chronic HBV mono-infected individuals
$I_{AC}$	Co-infected with acute HBV infection
$I_{CC}$	Co-infected with chronic HBV infection
$C_{R}$	COVID-19 recovery individuals
$H_{T}$	HBV treatment individuals

With the basic concepts illustrated by the individuals flow diagram given by Fig. 1 the HBV and COVID-19 co-epidemic dynamical system (model) is illustrated by

3 $$ \begin{aligned} \dot{S} & = k_{1} {\Delta } + \alpha_{1} C_{P} + \alpha_{2} H_{P} + {\uprho }C_{V} + {\upeta }C_{R} - \left( {\lambda_{H} + \lambda_{C} + \mu } \right)S, \\ \dot{C}_{P} & = k_{2} {\Delta } - \left( {\lambda_{H} + \alpha_{1} + \mu } \right)C_{P} , \\ \dot{H}_{P} & = k_{3} {\Delta } - \left( {\alpha_{2} + \mu + \lambda_{C} } \right)H_{P} , \\ \dot{C}_{V} & = k_{4} {\Delta } - \left( {\rho + \mu + \lambda_{H} + \varepsilon \lambda_{C} } \right)C_{V} , \\ \dot{C}_{I} & = \lambda_{C} S + \lambda_{C} H_{P} + \varepsilon \lambda_{C} C_{V} - \left( {\mu + d_{1} + \kappa + \upsilon \lambda_{H} } \right)C_{V} , \\ \dot{H}_{A} & = \lambda_{H} S + \lambda_{H} C_{P} + \lambda_{H} C_{V} + {\uptheta }_{1} I_{AC} - \left( {{\uptheta } + \mu + \phi_{1} \lambda_{C} } \right)H_{A} , \\ \dot{H}_{C} & = {\uptheta }H_{A} + {\uptheta }_{2} I_{CC} - \left( {\gamma + d_{2} + \mu + \phi_{2} \lambda_{C} } \right)H_{C} , \\ \mathop {I_{AC} }\limits^{.} & = \phi_{1} \lambda_{C} H_{A} + \upsilon \lambda_{H} C_{I} - \left( {\mu + d_{3} + \delta + {\uptheta }_{1} } \right)I_{AC} , \\ \mathop {I_{CC} }\limits^{.} & = \delta I_{AC} + \phi_{2} \lambda_{C} H_{C} - \left( {\mu + d_{4} + {\uptheta }_{2} } \right)I_{CC} , \\ \dot{C}_{R} & = \kappa C_{I} - \left( {\mu + \eta } \right)C_{R} , \\ \dot{H}_{T} & = \gamma H_{C} - \mu H_{T} , \\ \end{aligned} $$

with initial data given by

4 $$ \begin{gathered} S\left( 0 \right) > 0,\;C_{P} \left( 0 \right) \ge 0, H_{P} \left( 0 \right) \ge 0, C_{V} \left( 0 \right) \ge 0,\;C_{I} \left( 0 \right) \ge 0,H_{A} \left( 0 \right) \ge 0,\; H_{C} \left( 0 \right) \ge 0,I_{AC} \left( 0 \right) \ge 0,\;I_{CC} \left( 0 \right) \ge 0,\; \hfill \\ C_{R} > 0,\;{\text{and}}\;H_{T} > 0. \hfill \\ \end{gathered} $$

The derivative for the total population using (3) is computed as

5 $$ \dot{N} = {\Delta } - \mu N - \left( {d_{1} C_{I} + d_{2} H_{C} + d_{3} I_{AC} + d_{4} I_{CC} } \right) $$

### The model solutions non-negativity and boundedness

The HBV and COVID-19 co-epidemic dynamical system (3) is epidemiologically meaningful whenever each of the co-epidemic dynamical system solutions becomes non-negative and also bounded in space region illustrated by

6 $$ {\Omega } = \left\{ {\begin{array}{*{20}c} {\left( {S,C_{P} ,H_{P} , C_{V} ,C_{I} ,H_{A} ,H_{C} ,I_{AC} ,I_{CC} ,C_{R} ,H_{T} } \right) \in {\mathbb{R}}_{ + }^{11} ,N \le \frac{{\Lambda }}{\mu }} \\ \end{array} } \right\}. $$

#### Theorem 1 (Non-negativity):

The co-epidemic dynamical system (3) solutions defined by $$ S\left( t \right)$$, $$C_{P} \left( t \right),$$ $$ H_{P} \left( t \right), C_{V} \left( t \right), C_{I} \left( t \right), H_{A} \left( t \right)$$, $$H_{C} \left( t \right)$$, $$I_{AC} \left( t \right)$$, $$ I_{CC} \left( t \right),$$ $$C_{R} \left( t \right), $$ and $$ H_{T} \left( t \right)$$ with the initial conditions described in (4) are non-negative for any arbitrary time $$ t > 0$$.

#### Proof:

Let us consider the initial population as $S\left( 0 \right) > 0$,$C_{P} \left( 0 \right) > 0,H_{P} \left( 0 \right) > 0$,$C_{V} \left( 0 \right) > 0$,$C_{I} \left( 0 \right) > 0$,$H_{P} \left( 0 \right) > 0$,$H_{C} \left( 0 \right) > 0$,$I_{AC} \left( 0 \right) > 0, I_{CC} \left( 0 \right) > 0$,$C_{R} \left( 0 \right) > 0,$ and $H_{T} \left( 0 \right) > 0$ then for all time t > 0, we have to prove that $S$ (t) > 0,$C_{P} \left( t \right) > 0,H_{P} \left( t \right) > 0$,$C_{V} \left( t \right)$ > 0, $C_{I} \left( t \right)$ > 0, $H_{A} \left( t \right)$ > 0, $H_{C} \left( t \right)$ > 0, $I_{AC} \left( t \right)$ > 0, $I_{CC} \left( t \right)$ > 0, $C_{R} \left( t \right) > 0,$ and $H_{T} \left( t \right)$ > 0.

Define:$\tau$ = sup $\{ t > 0:S{ }\left( {\text{t}} \right){ } > 0, C_{P} \left( t \right) > 0, H_{P} \left( t \right) > 0$,$C_{V} \left( t \right) > { }0, C_{I} \left( t \right) > 0$,$H_{A} \left( t \right)$ > 0,$H_{C} \left( t \right)$ > 0, $I_{AC} \left( t \right)$ > 0, $I_{CC} \left( t \right)$ > 0$, C_{R} \left( t \right) > { }0, {\text{and }}H_{T} \left( t \right) > { }0\}$. Since all the HBV and COVID-19 co-epidemic state variables $S\left( t \right), C_{P} \left( t \right)$,$H_{P} \left( t \right),C_{V} \left( t \right), C_{I} \left( t \right), H_{A} \left( t \right),$ $H_{C} \left( t \right), I_{AC} \left( t \right)$,$I_{CC} \left( t \right),C_{R} \left( t \right),$ and $H_{T} \left( t \right)$ are continuous we can justify that $\tau > 0$. If $\tau$ =  + ∞, then non-negativity holds. But, if 0 < $\tau$ <  + ∞ we will have $S\left( \tau \right) = 0,$ or $C_{P} \left( \tau \right) = 0,$ or $H_{P} \left( \tau \right) = 0,$ or $C_{P} \left( \tau \right) = 0,$ or $C_{V} \left( { \tau { }} \right) = 0,$ or $H_{A} \left( { \tau { }} \right) = 0,$ or $H_{C} \left( { \tau { }} \right) = 0,$ or $I_{AC} \left( { \tau { }} \right) = 0,$ or $I_{CC} \left( { \tau { }} \right) = 0,$ or $C_{R} \left( { \tau { }} \right) = 0,$ or $H_{T} \left( { \tau { }} \right) = 0.$

From the first equation of the full model (3) we do have

$$ \dot{S} + \left( {\lambda_{H} + \lambda_{C} + \mu } \right)S = k_{1} {\Delta } + \alpha_{1} C_{P} + \alpha_{2} H_{P} + {\uprho }C_{V} + {\upeta }C_{V} . $$

And integrate both sides using integrating factor we have determined the constant value.

$S\left( \tau \right) = M_{1} S\left( 0 \right)$ + $M_{1} \mathop \smallint \limits_{0}^{\tau } exp^{{\smallint \left( {\mu + \lambda_{H} \left( t \right) + \lambda_{C} \left( t \right)} \right)dt}} \left( {k_{1} {\Delta } + \alpha_{1} C_{P} + \alpha_{2} H_{P} + {\uprho }C_{V} + {\upeta }C_{R} } \right)dt > 0$ where $M_{1} = exp^{{ - \left( {\mu \tau + \mathop \smallint \limits_{0}^{\tau } (\lambda_{H} \left( w \right) + \lambda_{C} \left( w \right)} \right)}} > 0, S\left( 0 \right) > 0,$ and from the meaning of $\tau ,$ the solutions $C_{P} \left( t \right) > 0,$ $H_{P} \left( t \right) > 0, C_{V} \left( t \right) > 0,$ $C_{R} \left( t \right) > 0,$ also the exponential function always is positive, then the solution $S\left( \tau \right) > 0$ hence $S\left( \tau \right) \ne 0$.

Again from the second equation of the full model (3) we do have

$$ \dot{C}_{P} + \left( {\lambda_{H} + \alpha_{1} + \mu } \right)C_{P} = k_{2} {\Delta }{.} $$

And also using t integrating factor after some calculations we obtained that

$C_{P} \left( \tau \right) = M_{1} C_{P} \left( 0 \right) + M_{1} \mathop \smallint \limits_{0}^{\tau } exp^{{\smallint \left( {\lambda_{H} + \alpha_{1} + \mu )} \right)dt}} k_{2} {\Delta }dt > 0$ where $M_{1} = exp^{{ - \left( {\alpha_{1} \tau + \mu \tau + \mathop \smallint \limits_{0}^{\tau } (\lambda_{H} \left( w \right)} \right)}} > 0, C_{P} \left( 0 \right) > 0,$ and from the meaning of $\tau ,$ the solution $C_{P} \left( \tau \right) > 0$ hence $C_{P} \left( \tau \right) \ne 0$.

Similarly, $H_{P} \left( \tau \right) > 0,$ hence $H_{P} \left( \tau \right) \ne 0$, $C_{V} \left( \tau \right) > 0,$ hence $C_{V} \left( \tau \right) \ne 0$, $C_{I} \left( \tau \right) > 0,$ hence $C_{I} \left( \tau \right) \ne 0$, $H_{A} \left( \tau \right) > 0,$ hence $H_{A} \left( \tau \right) \ne 0$, $H_{C} \left( \tau \right) > 0$ hence $H_{C} \left( \tau \right) \ne 0$, $I_{AC} \left( \tau \right) > 0,$ hence $I_{AC} \left( \tau \right) \ne 0$, $I_{CC} \left( \tau \right) > 0$, hence $I_{CC} \left( \tau \right) \ne 0$, $C_{R} \left( \tau \right) > 0,$ hence $C_{R} \left( \tau \right) \ne 0$, and $H_{T} \left( \tau \right) > 0,$ hence $H_{T} \left( \tau \right) \ne 0$.

Thus, $\tau = + \infty$, and hence all the solutions of the COVID-19 and HBV co-epidemic model (3) are non-negative.

#### Theorem 2 (Boundedness):

The HBV and COVID-19 co-epidemic model solutions are bounded in the region described in Eq. (6).

#### Proof:

Let $\left( {S,C_{P} ,H_{P} , C_{V} ,C_{I} ,H_{A} ,H_{C} ,I_{AC} ,I_{CC} ,C_{R} ,H_{T} } \right) \in {\mathbb{R}}_{ + }^{11}$ be an arbitrary non-negative solution of the system (3) with initial conditions given in Eq. (4).

Now adding all the differential equations given in Eq. (3) we have determined the derivative of the total population $N$ which is given in Eq. (5) as $\dot{N} = {\Delta } - \mu N - (d_{1} C_{I} + d_{2} H_{C} + d_{3} I_{AC} + d_{4} I_{CC} ).$ Then by ignoring all the infection classes we have determined that $\dot{N} \le {\Delta } - \mu N,$ and using separation of variables whenever $t \to \infty ,$ we have obtained that $0 \le N \le \frac{{\Delta }}{\mu }$. Hence, all the positive feasible non-negative solutions of the co-epidemic model (3) entering in to the region given in Eq. (6).

## Qualitative analysis of the dynamical system

Before investigating the qualitative aspects of the COVID-19 and HBV co-epidemic system (3), it is fundamental to collect some basic concepts about single infection sub-models of HBV or COVID-19.

### The HBV sub-model qualitative analysis

In this part by assuming the absence of COVID-19 infection as $C_{P} =$ $C_{V} =$ $C_{I} = I_{AC} = I_{CC} = C_{R} = 0$ in Eq. (3) then the HBV mono-infection system dynamics illustrated by

7 $$ \begin{aligned} \dot{S} & = k_{1} {\Delta } + \alpha_{2} H_{P} - \left( {\lambda_{H} + \mu } \right)S, \\ \dot{H}_{P} & = k_{3} {\Delta } - \left( {\alpha_{2} + \mu } \right)H_{P} , \\ \dot{H}_{A} & = \lambda_{H} S - \left( {{\uptheta } + \mu } \right)H_{A} , \\ \dot{H}_{C} & = {\uptheta }H_{A} - \left( {\gamma + d_{1} + \mu } \right)H_{C} , \\ \dot{H}_{T} & = \gamma H_{C} - \mu H_{T} , \\ \end{aligned} $$

such that $N_{1} \left( t \right) = S\left( t \right) + H_{P} \left( t \right) + H_{A} \left( t \right) + H_{C} \left( t \right) + H_{T} \left( t \right)$ is the total population, $\lambda_{H} = \frac{{\beta_{1} }}{{N_{1} }}\left( {H_{A} + \rho_{1} H_{C} } \right)$ is HBV infection rate at initial population illustrated $S\left( 0 \right) > 0, H_{P} \left( 0 \right) \ge 0$,$H_{A} \left( 0 \right) \ge 0$,$H_{C} \left( 0 \right) \ge 0$ and $H_{T} \left( 0 \right) \ge 0$.

Similarly the HBV sub-model (7) is epidemiologically meaningful in the space region illustrated by $${\Omega }_{1} = \left\{ {\begin{array}{*{20}c} {\left( {S,H_{P} ,H_{A} ,H_{C} ,H_{T} } \right) \in {\mathbb{R}}^{5}_{ + } ,N_{1} \le \frac{{\Delta }}{\mu }} \\ \end{array} } \right\}$$.

#### Local stability of HBV disease-free equilibrium

To compute the HBV sub-model (7) HBV disease-free equilibrium point make its right-hand equation as zero and putting $H_{A} = H_{C} = H_{T} = 0$ we determined the results $S^{0} = \frac{{k_{1} {\Delta }\left( {\alpha_{2} + \mu } \right) + \alpha_{2} k_{3} {\Delta }}}{{\mu \left( {\alpha_{2} + \mu } \right)}}$, $H_{P}^{0} = \frac{{k_{3} {\Delta }}}{{\alpha_{2} + \mu }}.$ Thus, the HBV sub-model (7) HBV disease-free equilibrium is represented by $E_{HM}^{0} = \left( {S^{0} ,H_{P}^{0} ,0,0,0} \right) = \left( {\frac{{k_{1} {\Delta }\left( {\alpha_{2} + \mu } \right) + \alpha_{2} k_{3} {\Delta }}}{{\mu \left( {\alpha_{2} + \mu } \right)}},\frac{{k_{3} {\Delta }}}{{\alpha_{2} + \mu }},0,0,0} \right)$.

The HBV equilibrium point $$E_{HM}^{0} $$ local stability is investigated by analyzing the sub-model reproduction number $${ \mathcal{R}}_{HM}$$ calculated by the approach stated in.^32^ Using the same approach stated in^32^ we derived the HBV sub-model reproduction number by the expression given by

$$ { \mathcal{R}}_{HM} = \frac{{\beta_{1} \left( {1 - k_{3} } \right)\left( {\alpha_{2} + \mu } \right) + \beta_{1} \alpha_{2} k_{3} }}{{\left( {\alpha_{2} + \mu } \right)\left( {\theta + \mu + d_{2} } \right)}} + \frac{{\beta_{1} \rho_{1} \theta \left( {1 - k_{3} } \right)\left( {\alpha_{2} + \mu } \right) + \beta_{1} \rho_{1} \theta \alpha_{2} k_{3} }}{{\left( {\theta + \mu + d_{2} } \right)\left( {\gamma + \mu + d_{3} } \right)}}. $$

##### Theorem 3

The HBV disease-free equilibrium point of the sub-model (7) is locally asymptotically stable if ${ \mathcal{R}}_{HM} < 1$, otherwise unstable.

##### Proof

To prove the locally asymptotic stability of the HBV disease-free equilibrium point, we can apply the criteria derived by Routh-Hurwitz.^53^

The associated Jacobian matrix of the sub-model (7) at the given equilibrium point $E_{HM}^{0}$ is illustrated by

$$ J\left( {E_{HM}^{0} } \right) = \left[ {\begin{array}{*{20}c} { - \mu } & {\alpha_{2} } & { - \frac{{\beta_{1} S^{0} }}{{S^{0} + H_{P}^{0} }}} & { - \frac{{\beta_{1} \rho_{1} S^{0} }}{{S^{0} + H_{P}^{0} }}} & 0 \\ 0 & { - \left( {\alpha_{2} + \mu } \right)} & 0 & 0 & 0 \\ 0 & 0 & {\frac{{\beta_{1} S^{0} }}{{S^{0} + H_{P}^{0} }} - \left( {{\uptheta } + \mu + d_{2} } \right)} & {\frac{{\beta_{1} \rho_{1} S^{0} }}{{S^{0} + H_{P}^{0} }}} & 0 \\ 0 & 0 & {\uptheta } & { - \left( {\gamma + d_{3} + \mu } \right)} & 0 \\ 0 & 0 & 0 & \gamma & { - \mu } \\ \end{array} } \right], $$

To derive the associated characteristic polynomial, let us compute the equation represented by

$$ \left| {\begin{array}{*{20}c} { - \mu - \lambda } & {\alpha_{2} } & { - \frac{{\beta_{1} S^{0} }}{{S^{0} + H_{P}^{0} }}} & { - \frac{{\beta_{1} \rho_{1} S^{0} }}{{S^{0} + H_{P}^{0} }}} & 0 \\ 0 & { - \left( {\alpha_{2} + \mu } \right) - \lambda } & 0 & 0 & 0 \\ 0 & 0 & {\frac{{\beta_{1} S^{0} }}{{S^{0} + H_{P}^{0} }} - \left( {{\uptheta } + \mu + d_{2} } \right) - \lambda } & {\frac{{\beta_{1} \rho_{1} S^{0} }}{{S^{0} + H_{P}^{0} }}} & 0 \\ 0 & 0 & {\uptheta } & { - \left( {\gamma + d_{3} + \mu } \right) - \lambda } & 0 \\ 0 & 0 & 0 & \gamma & { - \mu - \lambda } \\ \end{array} } \right| = 0. $$

$$ \begin{gathered} \Rightarrow \left( { - \mu - \lambda } \right)\left( { - \left( {\alpha_{2} + \mu } \right) - \lambda } \right)\left( { - \mu - \lambda } \right) \left[ {\left( {\frac{{\beta_{1} S^{0} }}{{S^{0} + H_{P}^{0} }} - \left( {\theta + \mu + d_{22} } \right) - \lambda } \right)} \right.\left( { - \left( {\gamma + d_{3} + \mu } \right) - \lambda } \right) \hfill \\ \left. {\frac{{\beta_{1} \rho_{1} S^{0} }}{{S^{0} + H_{P}^{0} }}} \right] = 0. \hfill \\ \end{gathered} $$

Simplifying the last expression we have $\left( { - \mu - \lambda } \right)\left( { - \left( {\alpha_{2} + \mu } \right) - \lambda } \right)\left( { - \mu - \lambda } \right)\left( {\lambda^{2} + a\lambda + b} \right) = 0$, where

$$ a = \left( {\gamma + d_{3} + \mu } \right) + \left( {\theta + \mu + d_{2} } \right) - \frac{{\beta_{1} S^{0} }}{{S^{0} + H_{P}^{0} }}, $$

and

$$ b = \left( {\theta + \mu + d_{2} } \right)\left( {\gamma + d_{3} + \mu } \right)\left( {1 - \frac{{\beta_{1} \rho_{1} \theta \left( {1 - k_{3} } \right)\left( {\alpha_{2} + \mu } \right) + \beta_{1} \rho_{1} \theta \alpha_{2} k_{3} }}{{\left( {\theta + \mu + d_{2} } \right)\left( {\gamma + \mu + d_{3} } \right)}}} \right) = \left( {\theta + \mu + d_{2} } \right)\left( {\gamma + d_{3} + \mu } \right)\left( {1 - { \mathcal{R}}_{HM} } \right). $$

The final result gives us $\lambda_{1} = - \mu < 0$ or $\lambda_{2} = - \left( {\alpha_{2} + \mu } \right) < 0$ or $\lambda_{3} = - \mu < 0$ or

8 $$ \lambda^{2} + a\lambda + b = 0. $$

Using the criteria derived by Routh-Hurwitz^53^ the quadratic equation given in (8) has two negative eigenvalues whenever $${ \mathcal{R}}_{HM} < 1$$, and hence each eigenvalue of the result has negative real part implies that $$ E_{HM}^{0} $$ is locally asymptotically stable whenever $${ \mathcal{R}}_{HM} < 1$$. The Theorem 3 proof result indicates that HBV infection spreading can be minimized in the community whenever $$ { \mathcal{R}}_{HM} < 1,$$ and whenever the initial data for HBV sub-model (7) is near to the equilibrium point $$ E_{HM}^{0}$$.

#### HBV infection endemic equilibrium(s)

Let $E^{*} = \left( {S^{*} ,H_{P}^{*} ,H_{A}^{*} ,H_{C}^{*} ,H_{T}^{*} } \right)$ be the HBV sub-model endemic equilibrium point. Then we make the equations in the right hand side of the dynamical system (7) becomes zero and computed to get the results expressed by

9 $$ \begin{gathered} S^{*} = \frac{{\alpha_{2} k_{3} {\Delta } + k_{1} {\Delta }h_{1} }}{{h_{1} \left( {\lambda_{H}^{*} + \mu } \right)}},\;H_{P}^{*} = \frac{{k_{3} {\Delta }}}{{h_{1} }},\;H_{A}^{*} = \frac{{\alpha_{2} k_{3} {\Delta }\lambda_{H}^{*} + k_{1} {\Delta }h_{1} \lambda_{H}^{*} }}{{h_{1} h_{2} \left( {\lambda_{H}^{*} + \mu } \right)}}, \hfill \\ H_{C}^{*} = \frac{{\alpha_{2} k_{3} {{\Delta \Theta }}\lambda_{H}^{*} + k_{1} {{\Delta \theta }}h_{1} \lambda_{H}^{*} }}{{h_{1} h_{2} h_{3} \left( {\lambda_{H}^{*} + \mu } \right)}}\;{\text{and}}\;H_{T}^{*} = \frac{{k_{1} {{\Delta \theta }}\gamma h_{1} \lambda_{H}^{*} + \alpha_{2} k_{3} {{\Delta \theta }}\gamma \lambda_{H}^{*} }}{{\mu h_{1} h_{2} h_{3} \left( {\lambda_{H}^{*} + \mu } \right)}}, \hfill \\ \end{gathered} $$

where $$h_{1} = \left( {\alpha_{2} + \mu } \right)$$, $$h_{2} = \left( {{\uptheta } + \mu + d_{2} } \right)$$, and $$h_{3} = \left( {\gamma + d_{3} + \mu } \right)$$.

We make substitution of the expressions $H_{A}^{*}$ and $H_{C}^{*}$ stated in Eq. (9) at the HBV infection rate given by $\lambda_{H}^{*} = \frac{{\beta_{1} H_{A}^{*} + \beta_{1} \rho_{1} H_{C}^{*} }}{{S^{*} + H_{P}^{*} + H_{A}^{*} + H_{C}^{*} + H_{T}^{*} }}$. The we have computed and simplified it as $\lambda_{H}^{*} = \frac{{h_{4} \lambda_{H}^{*} }}{{h_{5} + h_{6} \lambda_{H}^{*} }}$ and gives the result

10 $$ \left( {h_{5} + h_{6} \lambda_{H}^{*} - h_{4} } \right)\lambda_{H}^{*} = 0. $$

The non-zero solutions of Eq. (10) is $\lambda_{H}^{*} = \frac{{h_{4} - h_{5} }}{{h_{6} }}$ where $h_{4} = \beta_{1} k_{1} {\Delta }h_{1} h_{3} h_{3} \mu$+ $\beta_{1} \alpha_{2} k_{3} {\Delta }h_{3} h_{3} \mu$  + $\beta_{1} \rho_{1} k_{1} {{\Delta \theta }}h_{1} h_{3} \mu + \beta_{1} \rho_{1} \alpha_{2} k_{3} {{\Delta \theta }}h_{3} \mu$,$h_{5} = k_{1} {\Delta }h_{1} h_{2} h_{3} \mu + \alpha_{2} k_{3} {\Delta }h_{2} h_{3} \mu + k_{3} {\Delta }h_{2} h_{3} \mu \mu$, $h_{6} = k_{3} {\Delta }h_{2} h_{3} \mu$+$k_{1} {\Delta }h_{1} h_{3} \mu + \alpha_{2} k_{3} {\Delta }h_{3} \mu + k_{1} {{\Delta \theta }}h_{1} \mu + \alpha_{2} k_{3} {{\Delta \theta }}\mu + k_{1} {{\Delta \theta }}\gamma h_{1} + \alpha_{2} k_{3} {{\Delta \theta }}\gamma$.

Therefore, we derived the final result illustrated by

$$ \lambda_{H}^{*} = \frac{{\left[ {k_{1} {\Delta }m_{1} m_{2} m_{3} \mu + k_{3} {\Delta }m_{2} m_{3} \mu \left( {\alpha_{2} + \mu } \right)} \right]\left( {{ \mathcal{R}}_{HM} - 1} \right)}}{{k_{3} {\Delta }m_{2} m_{3} \mu + k_{1} {\Delta }m_{1} m_{3} \mu + \alpha_{2} k_{3} {\Delta }m_{3} \mu + k_{1} {{\Delta \theta }}m_{1} \mu + \alpha_{2} k_{3} {{\Delta \theta }}\mu + k_{1} {{\Delta \theta }}\gamma m_{1} + \alpha_{2} k_{3} {{\Delta \theta }}\gamma }}. $$

From this last expression, we have $\lambda_{H}^{*} > 0$ whenever ${\mathcal{R}}_{HM} > 1$ and hence the HBV sub-model (7) has a unique positive HBV infection endemic equilibrium point whenever ${\mathcal{R}}_{HM} > 1.$

##### Theorem 4:

The HBV dynamical system (7) has a unique positive HBV endemic equilibrium point whenever ${\mathcal{R}}_{HM} > 1$.

### Qualitative investigation of COVID-19 system dynamics

The associated COVID-19 sub-dynamical system of the complete co-epidemic model (3) is determined by making $H_{P} =$ $H_{A} = H_{C} = I_{AC} = I_{CC}$=$H_{T} = 0$, and it is represented by

11 $$ \begin{gathered} \dot{S} = k_{1} {\Delta } + \alpha_{1} C_{P} + {\uprho }C_{V} + {\upeta }C_{R} - \left( {\lambda_{C} + \mu } \right)S, \hfill \\ \dot{C}_{P} = k_{2} {\Delta } - \left( {\alpha_{1} + \mu } \right)C_{P} , \hfill \\ \dot{C}_{V} = k_{4} {\Delta } - \left( {\rho + \mu + \varepsilon \lambda_{C} } \right)C_{V} , \hfill \\ \dot{C}_{I} = \lambda_{C} S + \varepsilon \lambda_{C} C_{V} - \left( {\mu + d_{1} + \kappa } \right)C_{I} , \hfill \\ \dot{C}_{R} = \kappa C_{I} - \left( {\mu + \eta } \right)C_{R} , \hfill \\ \end{gathered} $$

At the initial population $S\left( 0 \right) > 0$, $C_{P} \left( 0 \right) \ge 0, C_{V} \left( 0 \right) \ge 0,$ $C_{I} \left( 0 \right) \ge 0$, $C_{R} \left( 0 \right) \ge 0$, total population represented by $N_{2} \left( t \right) = S\left( t \right) + C_{P} \left( t \right)$+$C_{V} \left( t \right) + C_{I} \left( t \right) + C_{R} \left( t \right),$ and COVID-19 force of infection given by $\lambda_{C} = \beta_{2} C_{I} \left( t \right)$.

#### Local stability of COVID-19 disease-free equilibrium point

By putting $C_{I} = C_{R} = 0$ for the system dynamics (11) we computed for the COVID-19 disease-free equilibrium point and simplifying the result we derived the results given by $S^{0} = \frac{{k_{1} {\Delta }(\alpha_{1} + \mu )\left( {\rho + \mu } \right) + \alpha_{1} k_{2} {\Delta }\left( {\rho + \mu } \right) + k_{4} {{\Delta \rho }}\left( {\alpha_{1} + \mu } \right)}}{{\mu \left( {\alpha_{1} + \mu } \right)\left( {\rho + \mu } \right)}}$, $C_{P}^{0} = \frac{{k_{2} {\Delta }}}{{\alpha_{1} + \mu }}$, and $C_{V}^{0} = \frac{{k_{4} {\Delta }}}{\rho + \mu }$. Thus, the COVID-19 disease-free equilibrium for the system (11) is represented by.

$$ E_{CM}^{0} = \left( {S^{0} ,C_{P}^{0} ,C_{V}^{0} ,C_{I}^{0} ,C_{R}^{0} } \right) = \left( {\frac{{k_{1} {\Delta }(\alpha_{1} + \mu )\left( {\rho + \mu } \right) + \alpha_{1} k_{2} {\Delta }\left( {\rho + \mu } \right) + k_{4} {{\Delta \rho }}\left( {\alpha_{1} + \mu } \right)}}{{\mu \left( {\alpha_{1} + \mu } \right)\left( {\rho + \mu } \right)}},\frac{{k_{2} {\Delta }}}{{\alpha_{1} + \mu }}{ },\frac{{k_{4} {\Delta }}}{\rho + \mu },0,0{ }} \right). $$

Using the same approach used by references^6,32^ we computed and simplified to obtain the COVID-19 reproduction number represented by

$$ {\mathcal{R}}_{CM} = \frac{{\beta_{2} S^{0} + \varepsilon \beta_{2} C_{V }^{0} }}{{\mu + d_{1} + \kappa }} = \frac{{\beta_{2} k_{1} {\Delta }(\alpha_{1} + \mu )\left( {\rho + \mu } \right) + \beta_{2} \alpha_{1} k_{2} {\Delta }\left( {\rho + \mu } \right) + \beta_{2} k_{4} {{\Delta \rho }}\left( {\alpha_{1} + \mu } \right) + \beta_{2} \varepsilon k_{4} {\Delta }\mu \left( {\alpha_{1} + \mu } \right)}}{{\mu \left( {\alpha_{1} + \mu } \right)\left( {\rho + \mu } \right)\left( {\mu + d_{1} + \kappa } \right)}}. $$

##### Theorem 5:

The COVID-19 disease-free equilibrium point $E_{CM}^{0}$ becomes locally asymptotically stable whenever ${\mathcal{R}}_{CM} < 1$, otherwise it is unstable.

##### Proof:

Let $E_{CM}^{0} = \left( {\frac{{k_{1} {\Delta }(\alpha_{1} + \mu )\left( {\rho + \mu } \right) + \alpha_{1} k_{2} {\Delta }\left( {\rho + \mu } \right) + k_{4} {{\Delta \rho }}\left( {\alpha_{1} + \mu } \right)}}{{\mu \left( {\alpha_{1} + \mu } \right)\left( {\rho + \mu } \right)}},\frac{{k_{2} {\Delta }}}{{\alpha_{1} + \mu }}{ },\frac{{k_{4} {\Delta }}}{\rho + \mu },0,0{ }} \right)$ be the sub-model (11) disease-free equilibrium point. To prove its local stability let us apply Routh-Hurwitz stability conditions explained in.^6,53^ The Jacobian matrix of the system (11) is derived as:

$$ J\left( {E_{CM}^{0} } \right) = \left[ {\begin{array}{*{20}c} { - \mu } & {\alpha_{1} } & {\uprho } \\ 0 & { - \left( {\alpha_{1} + \mu } \right)} & 0 \\ 0 & 0 & { - \left( {\rho + \mu } \right)} \\ 0 & 0 & 0 \\ 0 & 0 & 0 \\ \end{array} \begin{array}{*{20}c} { - \beta_{2} S^{0} } & {\upeta } \\ 0 & 0 \\ { - \beta_{2} \varepsilon C_{V}^{0} } & 0 \\ {\beta_{2} S^{0} + \beta_{2} \varepsilon C_{V}^{0} - \left( {\mu + d_{1} + \kappa } \right)} & 0 \\ \kappa & { - \left( {\mu + \eta } \right)} \\ \end{array} } \right]. $$

The equation derived from the matrix $J\left( {E_{CM}^{0} } \right)$ is the characteristics equation of the system (11) written as

$$ \left| {\begin{array}{*{20}c} { - \mu - \lambda } & {\alpha_{1} } & {\uprho } \\ 0 & { - \left( {\alpha_{1} + \mu } \right) - \lambda } & 0 \\ 0 & 0 & { - \left( {\rho + \mu } \right) - \lambda } \\ 0 & 0 & 0 \\ 0 & 0 & 0 \\ \end{array} \begin{array}{*{20}c} { - \beta_{2} S^{0} } & {\upeta } \\ 0 & 0 \\ { - \beta_{2} \varepsilon C_{V}^{0} } & 0 \\ {M - \lambda } & 0 \\ \kappa & { - \left( {\mu + \eta } \right) - \lambda } \\ \end{array} } \right| = 0, $$

where $M = \beta_{2} S^{0} + \beta_{2} \varepsilon C_{V}^{0} - \left( {\mu + d_{1} + \kappa } \right),$ and we calculated the eigenvalues given by $\lambda_{1} = - \mu < 0$ or $\lambda_{2} = - \left( {\alpha_{1} + \mu } \right) < 0$ or $\lambda_{3} = - \left( {\rho + \mu } \right) < 0$ or $\lambda_{4} = \beta_{2} S^{0} + \beta_{2} \varepsilon C_{V}^{0} - \left( {\mu + d_{1} + \kappa } \right) = \left( {\mu + d_{1} + \kappa } \right)\left[ {\frac{{\beta_{2} S^{0} + \beta_{2} \varepsilon C_{V}^{0} }}{{\mu + d_{1} + \kappa }} - 1} \right] = \mu + d_{1} + \kappa )\left[ {{\mathcal{R}}_{CM} - 1} \right] < 0$ whenever ${\mathcal{R}}_{CM} < 1$ or $\lambda_{5} = - \left( {\mu + \eta } \right) < 0.$

Thus, each of the system (11) eigenvalue has negative part whenever ${\mathcal{R}}_{CM} < 1$ and hence the results indicate that the COVID-19 disease-free equilibrium point $E_{CM}^{0}$ is locally asymptotically stable whenever ${\mathcal{R}}_{CM} < 1.$

#### Endemic equilibrium point(s) of the dynamical system (11)

Let $E_{C}^{*} = \left( {S^{*} ,C_{P}^{*} ,C_{V}^{*} ,C_{I}^{*} ,C_{R}^{*} } \right)$ becomes the COVID-19 endemic equilibrium point of the dynamical system (11) with the infection rate at the endemic equilibrium point given by $\lambda_{C}^{*} = \beta_{2} C_{I}^{*}$ and putting its right-hand sides equal to zero gives the final result given by

$$ S^{*} = \frac{{b_{5} \left( {b_{2} + \varepsilon \lambda_{C}^{*} } \right)^{2} + b_{6} \left( {b_{2} + \varepsilon \lambda_{C}^{*} } \right)^{2} + b_{7} \left( {b_{2} + \varepsilon \lambda_{C}^{*} } \right) + b_{8} \lambda_{C}^{*} }}{{b_{1} b_{3} b_{4} \left( {b_{2} + \varepsilon \lambda_{C}^{*} } \right)^{2} \left( {\lambda_{C}^{*} + \mu } \right) - b_{1} {\upeta }\kappa \left( {b_{2} + \varepsilon \lambda_{C}^{*} } \right)^{2} \lambda_{C}^{*} }}, $$

$$ C_{P}^{*} = \frac{{k_{2} {\Delta }}}{{b_{1} }},\;\;C_{V}^{*} = \frac{{k_{4} {\Delta }}}{{\left( {b_{2} + \varepsilon \lambda_{C}^{*} } \right)}}, $$

$$ \begin{aligned}   C_{V}^{*}  &  = \frac{{b_{5} \left( {b_{2}  + \varepsilon \lambda _{C}^{*} } \right)^{2} \lambda _{C}^{*}  + b_{6} \left( {b_{2}  + \varepsilon \lambda _{C}^{*} } \right)^{2} \lambda _{C}^{*}  + \left( {b_{2} b_{7} \lambda _{C}^{*}  + b_{7} \varepsilon \lambda _{C}^{{*2}} } \right)}}{{b_{{12}} \left( {b_{2}  + \varepsilon \lambda _{C}^{*} } \right)^{2} \left( {\lambda _{C}^{*}  + \mu } \right) - b_{{13}} \left( {b_{2}  + \varepsilon \lambda _{C}^{*} } \right)^{2} \lambda _{C}^{*} }} \\     & \;\;\; + \frac{{b_{8} \lambda _{C}^{{*2}}  + \left( {b_{2} b_{9}  + b_{9} \varepsilon \lambda _{C}^{*} } \right)\left( {\lambda _{C}^{{*2}}  + \mu \lambda _{C}^{*} } \right) - b_{{10}} \lambda _{C}^{{*2}}  - b_{{11}} \lambda _{C}^{{*3}} }}{{b_{{12}} \left( {b_{2}  + \varepsilon \lambda _{C}^{*} } \right)^{2} \left( {\lambda _{C}^{*}  + \mu } \right) - b_{{13}} \left( {b_{2}  + \varepsilon \lambda _{C}^{*} } \right)^{2} \lambda _{C}^{*} }}, \\  \end{aligned}  $$

and

$$ C_{R}^{*} = \frac{{\kappa D_{5}^{*} }}{{b_{4} }}, $$

where $$b_{1} = \alpha_{1} + \mu$$, $$b_{2} = \rho + \mu$$, $$b_{3} = \mu + d_{1} + \kappa$$, $$b_{4} = \mu + \eta$$, $$b_{5} = k_{1} {\Delta }b_{1} b_{3} b_{4}$$,$$b_{6} = \alpha_{1} k_{2} {\Delta }b_{3} b_{4}$$, $$b_{7} = {\uprho }k_{4} {\Delta }b_{1} b_{3} b_{4}$$, $$b_{8} = k_{4} {\Delta }b_{1} {\upeta }\kappa \varepsilon$$,$$b_{9} = b_{1} b_{3} b_{4} k_{4} {\Delta }\varepsilon$$, $$b_{10} = b_{2} b_{1} {\upeta }\kappa k_{4} {\Delta }\varepsilon$$, $$b_{11} = b_{1} k_{4} {\Delta }\varepsilon {\upeta }\kappa \varepsilon$$, $$b_{12} = b_{1} b_{3} b_{3} b_{4}$$, $$b_{13} = b_{1} b_{3} {\upeta }\kappa$$.

Then we substituted the result given by $C_{I}^{*}  = \frac{{b_{5} \left( {b_{2}  + \varepsilon \lambda _{C}^{*} } \right)^{2} \lambda _{C}^{*}  + b_{6} \left( {b_{2}  + \varepsilon \lambda _{C}^{*} } \right)^{2} \lambda _{C}^{*}  + \left( {b_{2} b_{7} \lambda _{C}^{*}  + b_{7} \varepsilon \lambda _{C}^{{*2}} } \right)}}{{b_{{12}} \left( {b_{2}  + \varepsilon \lambda _{C}^{*} } \right)^{2} \left( {\lambda _{C}^{*}  + \mu } \right) - b_{{13}} \left( {b_{2}  + \varepsilon \lambda _{C}^{*} } \right)^{2} \lambda _{C}^{*} }}$+ $\frac{{b_{8} \lambda _{C}^{{*2}}  + \left( {b_{2} b_{9}  + b_{9} \varepsilon \lambda _{C}^{*} } \right)\left( {\lambda _{C}^{{*2}}  + \mu \lambda _{C}^{*} } \right) - b_{{10}} \lambda _{C}^{{*2}}  - b_{{11}} \lambda _{C}^{{*3}} }}{{b_{{12}} \left( {b_{2}  + \varepsilon \lambda _{C}^{*} } \right)^{2} \left( {\lambda _{C}^{*}  + \mu } \right) - b_{{13}} \left( {b_{2}  + \varepsilon \lambda _{C}^{*} } \right)^{2} \lambda _{C}^{*} }},$ into $\lambda_{C}^{*} = \beta_{2} C_{I}^{*}$, and determined the polynomial

12 $$  f_{3} \lambda _{C}^{{*3}}  + f_{2} \lambda _{C}^{{*2}}  + f_{1} \lambda _{C}^{*}  + f_{0}  = 0,  $$

where

13 $$ f_{3} = b_{12} \varepsilon^{2} - b_{13} \varepsilon^{2} > 0,f_{2} = 2b_{2} b_{12} \varepsilon + b_{12} \mu \varepsilon^{2} - 2b_{2} b_{13} \varepsilon - b_{5} \varepsilon^{2} - b_{6} \varepsilon^{2} - b_{9} \varepsilon + b_{11} $$

$$f_{1} = b_{2}^{2} b_{12} + b_{10} + 2b_{2} b_{12} \varepsilon \mu - b_{2}^{2} b_{13} - 2b_{2} b_{5} \varepsilon - 2b_{2} b_{6} \varepsilon - b_{7} \varepsilon - b_{8} - b_{2} b_{9} - b_{9} \mu \varepsilon ,$$$$f_{0} = b_{1} b_{2} b_{3} b_{4} \left[ {1 - { \mathcal{R}}_{CM} } \right] > 0$$ Whenever $${\mathcal{R}}_{CM} < 1$$.

From Eq. (13) one can prove that $f_{3} > 0$ and $f_{0} > 0$ at ${\mathcal{R}}_{CM} < 1$ and the number of positive real solutions of (12) are based on the signs of the coefficients given by$f_{1}$, and$f_{2}$. Using the Descartes' criteria one can justify the number of positive solutions for the polynomial $f\left( y \right)$ = $f_{3} y^{3} + f_{2} y^{2} + f_{1} y + f_{0}$ (at $y$ = $\lambda_{C}^{*}$). Based on the Descartes' criteria we can state the following theorem.

##### Theorem 6:

The COVID-19 sub-dynamical system illustrated by (11) will have

- A unique positive COVID-19 endemic equilibrium point whenever ${\mathcal{R}}_{CM} > 1$ and either of the following conditions satisfies
  (i) $$f_{1} > 0$$ and $$f_{2} > 0$$.
  (ii) $$f_{1} < 0$$ and $$f_{2} < 0$$..

(b) More than one positive COVID-19 endemic equilibrium point whenever ${\mathcal{R}}_{CM} > 1$ and either of the following condition satisfies.
  (i) $$f_{1} > 0$$ and $$f_{2} < 0.$$
(ii) $$f_{1} < 0$$ and $$f_{2} > 0$$.

(c) Two positive COVID-19 endemic equilibrium points whenever ${\mathcal{R}}_{CM} < 1$, $f_{1} < 0$ and $f_{2} < 0$.

Using the criteria applied by references,^6,12,29,31,41,54^ item (c) of Theorem 6 indicates bifurcation of the dynamical system (11) in the backward direction whenever${ \mathcal{R}}_{CM} < 1$. The basic requirement having the sub-model (11) effective reproduction number ${\mathcal{R}}_{CM} < 1 ,$ although is necessary, is not sufficiently enough to the complete control of the COVID-19 infection spreading in the community.

##### Theorem 7:

The COVID-19 sub-model (11) reveals that the backward bifurcation at ${\mathcal{R}}_{CM} = 1$ whenever $H_{2} > H_{1}$ such that $H_{1}$ = $\frac{{ - \beta_{2} \beta^{*} x_{1}^{0} \left( {\rho + \mu } \right)\left( {\mu + \eta } \right) - \beta_{2} \beta^{*} \varepsilon x_{3}^{0} {\uprho }\left( {\mu + \eta } \right) - \beta_{2} \varepsilon \beta^{*} \varepsilon x_{3}^{0} \mu \left( {\mu + \eta } \right)}}{{\mu \left( {\rho + \mu } \right)\left( {\mu + \eta } \right)}}$, and $H_{2} = \frac{{\beta_{2} \kappa {\upeta }\left( {\rho + \mu } \right)}}{{\mu \left( {\rho + \mu } \right)\left( {\mu + \eta } \right)}}$.

##### Proof:

In this section, we apply the center manifold theory described in^29^ to ascertain the local stability of the endemic equilibrium due to the convolution of the first approach (eigenvalues of the Jacobian matrix). To make use of the center manifold theory, the following change of variables is made by symbolizing $S = x_{1}$,$C_{P} = x_{2} ,C_{V} = x_{3}$, $C_{I} = x_{4}$, and $C_{R} = x_{5}$ such that $N_{2} = x_{1} + x_{2} + x_{3} + x_{4} + x_{5}$. Furthermore, by using vector notation $X = \left( {x_{1} ,x_{2} ,x_{3} ,x_{4} ,x_{5} } \right)^{T}$, the COVID-19 mono-infection model (11) can be written in the form $\frac{dX}{{dt}} = F\left( X \right)$ with $F = \left( {f_{1} ,f_{2} ,f_{3} ,f_{4} ,f_{5} } \right)^{T}$, as follows

14 $$ \begin{gathered} \frac{{dx_{1} }}{dt} = f_{1} = k_{1} {\Delta } + \alpha_{1} x_{2} + {\uprho }x_{3} + {\upeta }x_{5} - \mu x_{1} - \lambda_{C} x_{1} , \hfill \\ \frac{{dx_{2} }}{dt} = f_{2} = k_{2} {\Delta } - \left( {\alpha_{1} + \mu } \right)x_{2} , \hfill \\ \frac{{dx_{3} }}{dt} = f_{4} = k_{4} {\Delta } - \left( {\rho + \mu + \varepsilon \lambda_{C} } \right)x_{3} , \hfill \\ \frac{{dx_{4} }}{dt} = \lambda_{C} x_{1} + \varepsilon \lambda_{C} x_{3} - \left( {\mu + d_{1} + \kappa } \right)x_{4} , \hfill \\ \frac{{dx_{5} }}{dt} = \kappa x_{4} - \left( {\mu + \eta } \right)x_{5} , \hfill \\ \end{gathered} $$

where $\lambda_{C} = \beta_{2} x_{5} .$ Then the method entails evaluating the Jacobian matrix of the system (14) at the DFE point$E_{CM}^{0}$, denoted by $J\left( {E_{CM}^{0} } \right)$ and it is computed as

$$ J\left( {E_{CM}^{0} } \right) = \left( {\begin{array}{*{20}c} { - \mu } & {\alpha_{2} } & {{{ \rho }}} & { - \beta_{2} x_{1}^{0} } & {\upeta } \\ 0 & {{ } - \left( {\alpha_{1} + \mu } \right)} & { } & 0 & 0 \\ {{ }0} & 0 & { - \left( {\rho + \mu } \right)} & { - \beta_{2} \varepsilon x_{3}^{0} } & 0 \\ 0 & 0 & 0 & {\beta_{2} x_{1}^{0} + \beta_{2} \varepsilon x_{3}^{0} - \left( {\mu + d_{1} + \kappa } \right)} & 0 \\ 0 & 0 & 0 & \kappa & { - \left( {\mu + \eta } \right)} \\ \end{array} } \right). $$

Consider, ${\mathcal{R}}_{CM} = 1$ and suppose $\beta_{2}$ $= \beta^{*}$ is chosen as a bifurcation parameter. From ${\mathcal{R}}_{CM} = 1$ as ${\mathcal{R}}_{CM} = \frac{{\beta_{2} x_{2}^{0} + \varepsilon \beta_{2} x_{4 }^{0} }}{{\mu + d_{1} + \kappa }} = \frac{{\beta_{2} k_{1} {\Delta }(\alpha_{1} + \mu )\left( {\rho + \mu } \right) + \beta_{2} \alpha_{1} k_{2} {\Delta }\left( {\rho + \mu } \right) + \beta_{2} k_{4} {\Delta }\left( {\alpha_{1} + \mu } \right)\left( {{\uprho } + \mu \varepsilon } \right)}}{{\mu \left( {\alpha_{1} + \mu } \right)\left( {\rho + \mu } \right)\left( {\mu + d_{1} + \kappa } \right)}} = 1$.

Solving for $\beta_{2}$ we have got $\beta_{2} = \beta^{*} = \frac{{\mu \left( {\alpha_{1} + \mu } \right)\left( {\rho + \mu } \right)\left( {\mu + d_{1} + \kappa } \right))}}{{k_{1} {\Delta }(\alpha_{1} + \mu )\left( {\rho + \mu } \right) + \alpha_{1} k_{2} {\Delta }\left( {\rho + \mu } \right) + k_{4} {\Delta }\left( {\alpha_{1} + \mu } \right)\left( {{\uprho } + \mu \varepsilon } \right)}}$.

$$ J_{{\beta^{*} }} = \left( {\begin{array}{*{20}c} { - \mu } & {\alpha_{2} } & {{{ \rho }}} & { - \beta^{*} x_{1}^{0} } & {\upeta } \\ 0 & {{ } - \left( {\alpha_{1} + \mu } \right)} & { } & 0 & 0 \\ {{ }0} & 0 & { - \left( {\rho + \mu } \right)} & { - \beta^{*} \varepsilon x_{3}^{0} } & 0 \\ 0 & 0 & 0 & {\beta^{*} x_{1}^{0} + \beta^{*} \varepsilon x_{3}^{0} - \left( {\mu + d_{1} + \kappa } \right)} & 0 \\ 0 & 0 & 0 & \kappa & { - \left( {\mu + \eta } \right)} \\ \end{array} } \right). $$

After some steps of the calculation we have computed the eigenvalues of $J_{{\beta^{*} }}$ as $\lambda_{1} = - \mu$, or $\lambda_{2} = - \left( {\alpha_{1} + \mu } \right),$ or or $\lambda_{3} = - \left( {\rho + \mu } \right),$ or $\lambda_{4} = 0,$ or $\lambda_{5} = - \left( {\mu + \eta } \right)$. It follows that the Jacobian matrix $J\left( {E_{CM}^{0} } \right)$ of Eq. (14) at the disease-free equilibrium point with $\beta_{2} = \beta^{*}$, denoted by $J_{{\beta^{*} }}$, has a single zero eigenvalue with all the remaining eigenvalues have negative real part. Hence, Theorem 2 of Castillo-Chavez and Song^29^ can be used to analyze the dynamics of the model to reveals that the model (11) undergoes backward bifurcation at ${\mathcal{R}}_{CM} = 1$.

Eigenvectors of $J_{{\beta^{*} }}$: For the case ${\mathcal{R}}_{CM} = 1$, it can be shown that the Jacobian of the system (14) at $\beta_{2} = \beta^{*}$ (denoted by $J_{{\beta^{*} }} )$ has a right eigenvectors associated with the zero eigenvalue given by $u = \left( {u_{1} ,u_{2} ,u_{3} ,u_{4} ,u_{5} } \right)^{T}$ as

15 $$ \left( {\begin{array}{*{20}c} { - \mu } & {\alpha_{2} } & {{{ \rho }}} & { - \beta^{*} x_{1}^{0} } & {\upeta } \\ 0 & {{ } - \left( {\alpha_{1} + \mu } \right)} & { } & 0 & 0 \\ {{ }0} & 0 & { - \left( {\rho + \mu } \right)} & { - \beta^{*} \varepsilon x_{3}^{0} } & 0 \\ 0 & 0 & 0 & {\beta^{*} x_{1}^{0} + \beta^{*} \varepsilon x_{3}^{0} - \left( {\mu + d_{1} + \kappa } \right)} & 0 \\ 0 & 0 & 0 & \kappa & { - \left( {\mu + \eta } \right)} \\ \end{array} } \right)\left( {\begin{array}{*{20}c} {u_{1} } \\ {u_{2} } \\ {u_{3} } \\ {u_{4} } \\ {u_{5} } \\ \end{array} } \right) = \left( {\begin{array}{*{20}c} 0 \\ 0 \\ 0 \\ 0 \\ 0 \\ \end{array} } \right). $$

Then solving Eq. (15) the right eigenvectors associated with the zero eigenvalue are given by

$$ u_{1} = \frac{{ - \beta^{*} x_{1}^{0} u_{4} \left( {\rho + \mu } \right)\left( {\mu + \eta } \right) - \beta^{*} \varepsilon x_{3}^{0} {\uprho }\left( {\mu + \eta } \right)u_{4} + \kappa {\upeta }\left( {\rho + \mu } \right)u_{4} }}{{\mu \left( {\rho + \mu } \right)\left( {\mu + \eta } \right)}}, $$

$$ u_{2} = 0,\;\;u_{3} = - \frac{{\beta^{*} \varepsilon x_{3}^{0} }}{{\left( {\rho + \mu } \right)}}u_{4} ,\;\;u_{4} = u_{4} > 0,\;u_{5} = \frac{\kappa }{\mu + \eta }u_{4} . $$

Similarly, the left eigenvector associated with the zero eigenvalues at $\beta_{2} = \beta^{*}$ given by $v = \left( {v_{1} ,v_{2} ,v_{3} ,v_{4} ,v_{5} } \right)^{T}$ as

16 $$ \left( {\begin{array}{*{20}c} {v_{1} } \\ {v_{2} } \\ {v_{3} } \\ {v_{4} } \\ {v_{5} } \\ \end{array} } \right)^{T} *\left( {\begin{array}{*{20}c} { - \mu } & {\alpha_{2} } & {{{ \rho }}} & { - \beta^{*} x_{1}^{0} } & {\upeta } \\ 0 & {{ } - \left( {\alpha_{1} + \mu } \right)} & { } & 0 & 0 \\ {{ }0} & 0 & { - \left( {\rho + \mu } \right)} & { - \beta^{*} \varepsilon x_{3}^{0} } & 0 \\ 0 & 0 & 0 & D & 0 \\ 0 & 0 & 0 & \kappa & { - \left( {\mu + \eta } \right)} \\ \end{array} } \right) = \left( {\begin{array}{*{20}c} 0 \\ 0 \\ 0 \\ 0 \\ 0 \\ 0 \\ \end{array} } \right), $$

where $$D = \beta^{*} x_{1}^{0} + \beta^{*} \varepsilon x_{3}^{0} - \left( {\mu + d_{1} + \kappa } \right)$$.

Then solving Eq. (16) the left eigenvectors associated with the zero eigenvalue are given by $v_{1} = v_{2} = v_{3} = v_{4} = 0$ and $v_{4} = v_{4} > 0$. After long steps of calculations the bifurcation coefficients $a$ and $b$ are obtained as

$$ \begin{aligned} a & = \mathop \sum \limits_{i,j,k = 1}^{5} v_{4} u_{i} u_{j} {\raise0.7ex\hbox{${\partial^{2} f_{4} }$} \!\mathord{\left/ {\vphantom {{\partial^{2} f_{4} } {\partial x_{i} \partial x_{j} }}}\right.\kern-0pt} \!\lower0.7ex\hbox{${\partial x_{i} \partial x_{j} }$}} = 2v_{4} u_{1} u_{4} {\raise0.7ex\hbox{${\partial^{2} f_{4} }$} \!\mathord{\left/ {\vphantom {{\partial^{2} f_{4} } {\partial x_{1} \partial x_{4} }}}\right.\kern-0pt} \!\lower0.7ex\hbox{${\partial x_{1} \partial x_{4} }$}} + 2v_{4} u_{3} u_{4} {\raise0.7ex\hbox{${\partial^{2} f_{4} }$} \!\mathord{\left/ {\vphantom {{\partial^{2} f_{4} } {\partial x_{3} \partial x_{4} }}}\right.\kern-0pt} \!\lower0.7ex\hbox{${\partial x_{3} \partial x_{4} }$}} \\ & = 2v_{4} u_{4} \left[ {u_{1} {\raise0.7ex\hbox{${\partial^{2} f_{4} }$} \!\mathord{\left/ {\vphantom {{\partial^{2} f_{4} } {\partial x_{1} \partial x_{4} }}}\right.\kern-0pt} \!\lower0.7ex\hbox{${\partial x_{1} \partial x_{4} }$}} + u_{3} {\raise0.7ex\hbox{${\partial^{2} f_{4} }$} \!\mathord{\left/ {\vphantom {{\partial^{2} f_{4} } {\partial x_{3} \partial x_{4} }}}\right.\kern-0pt} \!\lower0.7ex\hbox{${\partial x_{3} \partial x_{4} }$}}} \right], \\ & = 2v_{4} u_{4} \left[ {\beta_{2} u_{1} + \beta_{2} \varepsilon u_{3} } \right] \\ & = 2v_{4} u_{4}^{2} \left[ {\frac{{ - \beta_{2} \beta^{*} x_{1}^{0} \left( {\rho + \mu } \right)\left( {\mu + \eta } \right) - \beta_{2} \beta^{*} \varepsilon x_{3}^{0} {\uprho }\left( {\mu + \eta } \right) + \beta_{2} \kappa {\upeta }\left( {\rho + \mu } \right) - \beta_{2} \varepsilon \beta^{*} \varepsilon x_{3}^{0} \mu \left( {\mu + \eta } \right)}}{{\mu \left( {\rho + \mu } \right)\left( {\mu + \eta } \right)}}} \right], \\ & = 2v_{4} u_{4} \left[ {D_{2} - D_{1} } \right], \\ \end{aligned} $$

where $$H_{1}$$ = $$\frac{{ - \beta_{2} \beta^{*} x_{1}^{0} \left( {\rho + \mu } \right)\left( {\mu + \eta } \right) - \beta_{2} \beta^{*} \varepsilon x_{3}^{0} {\uprho }\left( {\mu + \eta } \right) - \beta_{2} \varepsilon \beta^{*} \varepsilon x_{3}^{0} \mu \left( {\mu + \eta } \right)}}{{\mu \left( {\rho + \mu } \right)\left( {\mu + \eta } \right)}}$$, and $$H_{2} = \frac{{\beta_{2} \kappa {\upeta }\left( {\rho + \mu } \right)}}{{\mu \left( {\rho + \mu } \right)\left( {\mu + \eta } \right)}}$$.

Thus, the bifurcation coefficient $a$ is positive whenever $D_{2} > D_{1}$.

Moreover

$$ b = \mathop \sum \limits_{i,k = 1}^{5} v_{k} u_{i} {\raise0.7ex\hbox{${\partial^{2} f_{k} }$} \!\mathord{\left/ {\vphantom {{\partial^{2} f_{k} } {\partial x_{i} \partial \beta }}}\right.\kern-0pt} \!\lower0.7ex\hbox{${\partial x_{i} \partial \beta }$}}\left( {E_{CM}^{0} } \right) = \mathop \sum \limits_{i = 1}^{5} v_{4} u_{i} {\raise0.7ex\hbox{${\partial^{2} f_{4} }$} \!\mathord{\left/ {\vphantom {{\partial^{2} f_{4} } {\partial x_{i} \partial \beta }}}\right.\kern-0pt} \!\lower0.7ex\hbox{${\partial x_{i} \partial \beta }$}} = v_{4} u_{4} {\raise0.7ex\hbox{${\partial^{2} f_{4} }$} \!\mathord{\left/ {\vphantom {{\partial^{2} f_{4} } {\partial x_{4} \partial \beta }}}\right.\kern-0pt} \!\lower0.7ex\hbox{${\partial x_{4} \partial \beta }$}} = v_{4} u_{4} \left[ {x_{1}^{0} u_{1} + \varepsilon x_{3}^{0} u_{3} } \right] > 0. $$

Hence, from Castillo-Chavez and Song^29^ the COVID-19 mono-infection model (11) exhibits a backward bifurcation at ${\mathcal{R}}_{CM} = 1$ and $H_{2} > H_{1}$.

### Qualitative investigation of the HBV and COVID-19 co-epidemic model

#### Disease-free equilibrium of the co-epidemic system

From the complete co-epidemic system (3) we compute the co-epidemic disease-free equilibrium by assuming the conditions $C_{I} = C_{R} = H_{A} = H_{C} = H_{T} = I_{AC} = I_{CC} = 0$ and after simplification we obtained the final result given by $E_{0} = \left( {S^{0} , C_{P}^{0} , H_{P}^{0} ,C_{V}^{0} , C_{I}^{0} , H_{A}^{0} ,H_{C}^{0} , I_{AC}^{0} , I_{CC}^{0} ,C_{R}^{0} , H_{T}^{0} } \right) = \left( {\frac{{k_{1} {\Delta }}}{\mu } + \frac{{\alpha_{1} k_{2} {\Delta }}}{{\alpha_{1} + \mu }} + \frac{{\alpha_{2} k_{3} {\Delta }}}{{\alpha_{2} + \mu }} + \frac{{{\uprho }k_{4} {\Delta }}}{\rho + \mu },\frac{{k_{2} {\Delta }}}{{\alpha_{1} + \mu }},\frac{{k_{3} {\Delta }}}{{\alpha_{2} + \mu }}, \frac{{k_{4} {\Delta }}}{\rho + \mu },0, 0, 0, 0, 0, 0, 0} \right)$.

#### The full co-epidemic model (3) effective reproduction number

Using a similar approach applied in “Local stability of HBV disease-free equilibrium” and “Local stability of COVID-19 disease-free equilibrium point” we computed the complete co-epidemic model (3) reproduction number illustrated by ${\mathcal{R}}_{HC} = {\text{max}}\left\{ {{ \mathcal{R}}_{CM} ,{ \mathcal{R}}_{HM} } \right\} = {\text{max}}\{ \frac{{\beta_{2} k_{1} {\Delta }(\alpha_{1} + \mu )\left( {\rho + \mu } \right) + \beta_{2} \alpha_{1} k_{2} {\Delta }\left( {\rho + \mu } \right) + \beta_{2} k_{4} {{\Delta \rho }}\left( {\alpha_{1} + \mu } \right) + \beta_{2} \varepsilon k_{4} {\Delta }\mu \left( {\alpha_{1} + \mu } \right)}}{{\mu \left( {\alpha_{1} + \mu } \right)\left( {\rho + \mu } \right)\left( {\mu + d_{1} + \kappa } \right)}}$, $\frac{{\beta_{1} \left( {1 - k_{3} } \right)\left( {\alpha_{2} + \mu } \right) + \beta_{1} \alpha_{2} k_{3} }}{{\left( {\alpha_{2} + \mu } \right)\left( {{\uptheta } + \mu + d_{2} } \right)}} + \frac{{\beta_{1} \rho_{1} {\uptheta }\left( {1 - k_{3} } \right)\left( {\alpha_{2} + \mu } \right) + \beta_{1} \rho_{1} {\uptheta }\alpha_{2} k_{3} }}{{\left( {{\uptheta } + \mu + d_{2} } \right)\left( {\gamma + \mu + d_{3} } \right)}}\} ,$ where ${\mathcal{R}}_{CM} = \frac{{\beta_{2} D_{1}^{0} + \varepsilon \beta_{2} D_{4 }^{0} }}{{\mu + d_{1} + \kappa }} = \frac{{\beta_{2} k_{1} {\Delta }(\alpha_{1} + \mu )\left( {\rho + \mu } \right) + \beta_{2} \alpha_{1} k_{2} {\Delta }\left( {\rho + \mu } \right) + \beta_{2} k_{4} {{\Delta \rho }}\left( {\alpha_{1} + \mu } \right) + \beta_{2} \varepsilon k_{4} {\Delta }\mu \left( {\alpha_{1} + \mu } \right)}}{{\mu \left( {\alpha_{1} + \mu } \right)\left( {\rho + \mu } \right)\left( {\mu + d_{1} + \kappa } \right)}}$ is the COVID-19 infection reproduction number and ${\mathcal{R}}_{HM} = \frac{{\beta_{1} \left( {1 - k_{3} } \right)\left( {\alpha_{2} + \mu } \right) + \beta_{1} \alpha_{2} k_{3} }}{{\left( {\alpha_{2} + \mu } \right)\left( {{\uptheta } + \mu + d_{2} } \right)}} + \frac{{\beta_{1} \rho_{1} {\uptheta }\left( {1 - k_{3} } \right)\left( {\alpha_{2} + \mu } \right) + \beta_{1} \rho_{1} {\uptheta }\alpha_{2} k_{3} }}{{\left( {{\uptheta } + \mu + d_{2} } \right)\left( {\gamma + \mu + d_{3} } \right)}}$ is the HBV only infection reproduction number.

#### Local stability of the full model (3) disease-free equilibrium

Using similar approach applied in,^6^ the Jacobian matrix of the complete HVB and COVID-19 co-epidemic is computed and written as

$$J\left({E}_{0}\right)=\left(\begin{array}{ccccccccccc}-\mu & {\alpha }_{1}& {\alpha }_{2}&\uprho & -{\upbeta }_{2}{S}^{0}& -\frac{{\beta }_{1}}{N}{S}^{0}& -\frac{{\beta }_{1}}{N}{S}^{0}{\rho }_{1}& -{{\text{D}}}_{4}& -{{\text{D}}}_{6}&\upeta & 0\\ 0& -\left({\alpha }_{1}+\mu \right)& 0& 0& 0& -\frac{{\beta }_{1}}{N}{C}_{P}^{0}& -\frac{{\beta }_{1}}{N}{D}_{2}^{0}{\rho }_{1}& -\frac{{\beta }_{1}}{N}{C}_{P}^{0}{\rho }_{2}& -\frac{{\beta }_{1}}{N}{C}_{P}^{0}{\rho }_{3}& 0& 0\\ 0& 0& -\left({\alpha }_{2}+\mu \right)& 0& -{\upbeta }_{2}{H}_{P}^{0}& 0& 0& -{\upbeta }_{2}{H}_{P}^{0}{\upomega }_{1}& -{\upbeta }_{2}{H}_{P}^{0}{\upomega }_{2}& 0& 0\\ 0& 0& 0& -\left(\rho +\mu \right)& -\varepsilon {\upbeta }_{2}{C}_{V}^{0}& -\frac{{\beta }_{1}}{{N}^{0}}{C}_{V}^{0}& -\frac{{\beta }_{1}}{{N}^{0}}{C}_{V}^{0}{\rho }_{1}& -{{\text{D}}}_{5}& -{{\text{D}}}_{7}& 0& 0\\ 0& 0& 0& 0& {{\text{D}}}_{3}& 0& 0& {\upomega }_{1}{{\text{D}}}_{3}& {\upomega }_{2}{{\text{D}}}_{3}& 0& 0\\ 0& 0& 0& 0& 0& {{\text{D}}}_{8}& {\rho }_{1}{{\text{D}}}_{8}& {\rho }_{2}{{\text{D}}}_{8}+{\uptheta }_{1}& {\rho }_{3}{{\text{D}}}_{8}& 0& 0\\ 0& 0& 0& 0& 0&\Theta & {{\text{D}}}_{9}& 0& {\uptheta }_{2}& 0& 0\\ 0& 0& 0& 0& 0& 0& 0& {{\text{D}}}_{10}& 0& 0& 0\\ 0& 0& 0& 0& 0& 0& 0& \delta & {{\text{D}}}_{11}& 0& 0\\ 0& 0& 0& 0& \kappa & 0& 0& 0& 0& -\left(\mu +\eta \right)& 0\\ 0& 0& 0& 0& 0& 0& \gamma & 0& 0& 0& -\mu \end{array}\right),$$

where $${\text{D}}_{3} = {\upbeta }_{2} \left( {S^{0} + H_{P}^{0} + \varepsilon C_{V}^{0} } \right) - \left( {\mu + d_{1} + \kappa } \right)$$, $${\text{D}}_{4} = \left( {\frac{{\beta_{1} }}{N}\rho_{2} + {\upbeta }_{2} {\upomega }_{1} } \right)S^{0}$$, $${\text{D}}_{5} = \left( {\frac{{\beta_{1} }}{{N^{0} }}\rho_{2} + \varepsilon {\upbeta }_{2} {\upomega }_{1} } \right)C_{V}^{0}$$, $${\text{D}}_{6} = \left( {\frac{{\beta_{1} }}{{N^{0} }}\rho_{3} + {\upbeta }_{2} {\upomega }_{2} } \right)S^{0}$$ and $${\text{D}}_{7} = \left( {\frac{{\beta_{1} }}{{N^{0} }}\rho_{3} + \varepsilon {\upbeta }_{2} {\upomega }_{2} } \right)C_{V}^{0}$$,$${\text{D}}_{8} = \frac{{\beta_{1} }}{N}\left( {S^{0} + C_{P}^{0} + C_{V}^{0} } \right) - \left( {{\uptheta } + \mu + d_{2} } \right)$$, $${\text{D}}_{9} = - \left( {\gamma + d_{3} + \mu } \right), {\text{D}}_{10} = - \left( {\mu + d_{4} + \delta + {\uptheta }_{1} } \right), {\text{D}}_{11} = - \left( {\mu + d_{5} + {\uptheta }_{2} } \right).$$

The corresponding eigenvalues of the matrix $J\left( {E_{0} } \right)$ are computed as $\lambda_{1} = - \mu < 0,$ or $\lambda_{2} = - \left( {\alpha_{1} + \mu } \right) < 0,$ or $\lambda_{3} = - \left( {\alpha_{2} + \mu } \right) < 0,$ or $\lambda_{4} = - \left( {\rho + \mu } \right) < 0,$ or $\lambda_{5} = - \mu < 0,$ or $\lambda_{6} = - \left( {\mu + \eta } \right) < 0,$ or $\lambda_{7} = \frac{{{\upbeta }_{2} \varepsilon k_{4} {\Delta }}}{{\left( {\rho + \mu } \right)\left( {\mu + d_{1} + \kappa } \right)}}\left( {{ \mathcal{R}}_{CM} - 1} \right) < 0$ or $\lambda_{8} = - \left( {\mu + d_{4} + \delta + {\uptheta }_{1} } \right) < 0,$ or $\lambda_{9} = - \left( {\mu + d_{5} + \Theta_{2} } \right) < 0,$ or $\lambda^{2} + \left[ {\left( {\gamma + d_{3} + \mu } \right) + \left( {{\uptheta } + \mu + d_{2} } \right) - D_{8} } \right]\lambda - \left[ {\left( {{\text{D}}_{8} - \left( {{\uptheta } + {\upmu } + {\text{d}}_{2} } \right)} \right)\left( {{\upgamma } + {\text{d}}_{3} + {\upmu }} \right) + {\uprho }_{1} {\text{D}}_{8} } \right]$ = 0.

Using the Routh-Hurwitz local stability conditions we can justify each of the given matrix eigenvalue has negative real part if ${\mathcal{R}}_{{{\text{HC}}}} = \max \left\{ {{ \mathcal{R}}_{CM} ,{ \mathcal{R}}_{HM} } \right\} < 1$ implies the result that co-epidemic disease-free equilibrium point has local asymptotic stability whenever ${\mathcal{R}}_{{{\text{HC}}}} = \max \left\{ {{ \mathcal{R}}_{CM} ,{ \mathcal{R}}_{HM} } \right\} < 1$.

## The optimal control problem and its qualitative analysis

This section aims to investigate the impacts of the time dependent optimal control measures on the HBV and COVID-19 co-epidemic spreading by applying Pontryagin’s Maximum Principle applied in references.^1,4,33,36^ Incorporating the four time dependent control strategies stated as: $0 \le {\text{w}}_{1} \left( {\text{t}} \right){ } \le 1$ becomes the HBV spreading protection measure, $0 \le {\text{w}}_{2} \left( {\text{t}} \right){ } \le 1$ becomes the COVID-19 spreading protection measure,$0 \le {\text{w}}_{3} \left( {\text{t}} \right){ } \le 1$ becomes the COVID-19 treatment measure, and $0 \le {\text{w}}_{4} \left( {\text{t}} \right){ } \le 1$ becomes the HBV treatment measure we re-formulate the full model (3) as

17 $$ \begin{gathered} \dot{S} = k_{1} {\Delta } + \alpha_{1} C_{P} + \alpha_{2} H_{P} + {\uprho }C_{V} + {\upeta }C_{R} - \left( {1 - w_{1} } \right)\lambda_{H} S - \left( {1 - w_{2} } \right)\lambda_{C} S - \mu S, \hfill \\ \dot{C}_{P} = k_{2} {\Delta } - \left( {1 - w_{1} } \right)\lambda_{H} C_{P} - \left( {\alpha_{1} + \mu } \right)C_{P} , \hfill \\ \dot{H}_{P} = k_{3} {\Delta } - \left( {1 - w_{2} } \right)\lambda_{C} H_{P} - \left( {\alpha_{2} + \mu } \right)H_{P} , \hfill \\ \dot{C}_{V} = k_{4} {\Delta } - \left( {1 - w_{1} } \right)\lambda_{H} C_{V} - \left( {1 - w_{2} } \right)\varepsilon \lambda_{C} C_{V} - \left( {\rho + \mu } \right)C_{V} , \hfill \\ \dot{C}_{I} = \left( {1 - w_{2} } \right)\lambda_{C} S + \left( {1 - w_{2} } \right)\lambda_{C} H_{P} + \left( {1 - w_{2} } \right)\varepsilon \lambda_{C} C_{V} - \left( {1 - w_{1} } \right)\upsilon \lambda_{H} C_{I} - \left( {\mu + d_{1} + {\mathfrak{u}}_{3} \kappa } \right)C_{I} , \hfill \\ \dot{H}_{A} = \left( {1 - w_{1} } \right)\lambda_{H} S + \left( {1 - w_{1} } \right)\lambda_{H} C_{P} + \left( {1 - w_{1} } \right)\lambda_{H} C_{V} + {\mathfrak{u}}_{3} {\uptheta }_{1} I_{AC} - \left( {1 - w_{2} } \right)\phi_{1} \lambda_{C} H_{A} - \left( {{\uptheta } + \mu + d_{2} } \right)H_{A} , \hfill \\ \dot{H}_{C} = {\uptheta }H_{A} + w_{3} {\uptheta }_{2} I_{CC} - \left( {1 - w_{2} } \right)\phi_{2} \lambda_{C} H_{C} - \left( {w_{4} \gamma + d_{3} + \mu } \right)H_{C} , \hfill \\ \mathop {I_{AC} }\limits^{.} = \left( {1 - w_{2} } \right)\phi_{1} \lambda_{C} H_{A} + \left( {1 - w_{1} } \right)\upsilon \lambda_{H} C_{I} - \left( {\mu + d_{4} + \delta + w_{3} {\uptheta }_{1} } \right)I_{AC} , \hfill \\ \mathop {I_{CC} }\limits^{.} = \delta I_{AC} + \left( {1 - w_{2} } \right)\phi_{2} \lambda_{C} H_{C} - \left( {\mu + d_{5} + w_{3} {\uptheta }_{2} } \right)I_{CC} , \hfill \\ \dot{C}_{R} = w_{3} \kappa C_{I} - \left( {\mu + \eta } \right)C_{R} , \hfill \\ \dot{H}_{T} = w_{4} \gamma H_{C} - \mu H_{T} , \hfill \\ \end{gathered} $$

With initial population ${\text{S}}\left( 0 \right) > 0$,${\text{C}}_{{\text{P}}} \left( 0 \right) \ge 0,{\text{ H}}_{{\text{P}}} \left( 0 \right) \ge 0,{\text{ C}}_{{\text{V}}} \left( 0 \right) \ge 0$,${\text{C}}_{{\text{I}}} \left( 0 \right) \ge 0,H_{A} \left( 0 \right) \ge 0$,

18 $$ H_{C} \left( 0 \right) \ge 0,I_{AC} \left( 0 \right) \ge 0,I_{CC} \left( 0 \right) \ge 0,\;C_{R} \left( 0 \right) > 0,\;{\text{and}}\;{\text{H}}_{T} \left( 0 \right) > 0. $$

The main objective of this optimal control problem is to determine the optimal controlling strategy ${\text{w}}^{*} = \left( {{\text{w}}_{1}^{*} ,{\text{ w}}_{2}^{*} ,{\text{w}}_{3}^{*} ,{\text{ w}}_{4}^{*} } \right)$ values for the control variables ${\text{w}} = \left( {{\text{w}}_{1} ,{\text{ w}}_{2} ,{\text{w}}_{3} ,{\text{ w}}_{4} } \right)$ such that $\left( {S^{*} ,{ }C_{P}^{*} ,{ }H_{P}^{*} ,C_{V}^{*} ,{ }C_{I}^{*} ,{ }H_{A}^{*} ,H_{C}^{*} ,{ }I_{AC}^{*} ,{ }I_{CC}^{*} ,C_{R}^{*} ,{ }H_{T}^{*} } \right)$ are solutions of the problem (17) in the time boundary $\left[ {0,{ }T_{f} } \right]$ at the initial population stated by (18) and the objective function represented by

19 $$ J\left( {{\text{w}}_{1} ,{\text{ w}}_{2} ,{\text{w}}_{3} ,{\text{ w}}_{4} } \right){ } = \mathop \smallint \limits_{0}^{{T_{f} }} \left( {{\upsigma }_{1} C_{I} + {\upsigma }_{2} { }H_{C} + {\upsigma }_{3} I_{AC} + {\upsigma }_{4} { }I_{CC} + \frac{{{\mathfrak{B}}_{1} }}{2}{\text{w}}_{1}^{2} + \frac{{{\mathfrak{B}}_{2} }}{2}{\text{w}}_{2}^{2} + \frac{{{\mathfrak{B}}_{3} }}{2}{\text{w}}_{3}^{2} + \frac{{{\mathfrak{B}}_{4} }}{2}{\text{w}}_{4}^{2} } \right)dt, $$

can be minimized with the associated coefficients given by $\sigma_{1} ,\sigma_{2} , \sigma_{3} ,$ and $\sigma_{4}$ and $\frac{{{\mathfrak{B}}_{1} }}{2}, \frac{{{\mathfrak{B}}_{2} }}{2}, \frac{{{\mathfrak{B}}_{3} }}{2},$ and $\frac{{{\mathfrak{B}}_{4} }}{2}$ are relative costs measure corresponding to $w_{1} , w_{2} ,w_{3}$ and $w_{4}$, in the stated order, and also it balances the given integrand. The term $\sigma_{1} C_{I}$ represents the cost associated with COVID-19 infectious group, the term $\sigma_{2} H_{C}$ represents the cost associated to chronic HBV infectious group, $\sigma_{3} I_{AC}$ represents the cost corresponding to the acute HBV and COVID-19 co-epidemic group and $\sigma_{4} I_{CC}$ represents the cost corresponding to chronic HBV and COVID-19 co-epidemic group.

$I\left( {S,{ }C_{P} { },H_{P} { },C_{V} ,{ }C_{I} ,{ }H_{A} ,{ }H_{C} ,{ }I_{AC} ,I_{CC} ,C_{R} ,{ }H_{T} ,{\mathfrak{u}}} \right){ } = {\upsigma }_{1} ,{ }C_{I} + {\upsigma }_{2} { }H_{C} + {\upsigma }_{3} I_{AC} + {\upsigma }_{4} { }I_{CC} + \frac{{{\mathfrak{B}}_{1} }}{2}{\text{w}}_{1}^{2} + \frac{{{\mathfrak{B}}_{2} }}{2}{\text{w}}_{2}^{2} + \frac{{{\mathfrak{B}}_{3} }}{2}{\text{w}}_{3}^{2} + \frac{{{\mathfrak{B}}_{4} }}{2}{\text{w}}_{4}^{2}$, investigates the cost at given time t. The collection of admissible Lebesgue measurable control functional is described as

20 $$ {\Omega }_{{\mathfrak{u}}} = \left\{ {\left( {{\text{w}}_{1} \left( t \right),{\text{ w}}_{2} \left( t \right),{\text{w}}_{3} \left( t \right),{\text{ w}}_{4} \left( t \right)} \right) \in L^{4} :0 \le {\text{w}}_{1} \left( t \right),{\text{ w}}_{2} \left( t \right),{\text{w}}_{3} \left( t \right),{\text{ w}}_{4} \left( t \right) \le 1,{ }t \in \left[ {0,T_{f} } \right]} \right\}. $$

Specifically, we need an optimal control minimum strategy written as

21 $$  J\left( {{\text{w}}_{1}^{{\text{*}}} ,{\text{~w}}_{2}^{{\text{*}}} ,{\text{w}}_{3}^{{\text{*}}} ,{\text{~w}}_{4}^{{\text{*}}} } \right) = \mathop {\min }\limits_{{{{\Omega }}_{u} }} J\left( {{\text{w}}_{1} ,{\text{~w}}_{2} ,{\text{w}}_{3} ,{\text{~w}}_{4} } \right). $$

### Theorems on the existence and uniqueness for optimal control problem

#### Theorem 9 (Existence of optimal control functions)

For the dynamical system (17) there exists an optimal control function ${\text{w}}^{*} = \left( {{\text{w}}_{1}^{*} ,{\text{ w}}_{2}^{*} ,{\text{w}}_{3}^{*} ,{\text{ w}}_{4}^{*} } \right)$ in the region ${\Omega }_{{\mathfrak{u}}}$ and the associated solution represented by $\left( {S^{*} ,{ }C_{P}^{*} ,{ }H_{P}^{*} ,C_{V}^{*} ,{ }C_{I}^{*} ,{ }H_{A}^{*} ,{ }H_{C}^{*} ,{ }I_{AC}^{*} ,{ }I_{CC}^{*} ,{ }C_{R}^{*} ,{ }H_{T}^{*} } \right)$ to the system dynamics (17) at the initial population stated in (18) as $J\left( {{\text{w}}_{1}^{*} ,{\text{ w}}_{2}^{*} ,{\text{w}}_{3}^{*} ,{\text{ w}}_{4}^{*} } \right) = \mathop {\min }\limits_{{{\Omega }_{{\mathfrak{u}}} }} J\left( {{\text{w}}_{1} ,{\text{ w}}_{2} ,{\text{w}}_{3} ,{\text{ w}}_{4} } \right)$.

#### Remark:

For the qualitative analysis of the dynamical system stated in (17) we applied the Pontryagin's Maximal principle used by scholars of the references.^1,4,33,36^

The Hamiltonian function for the system dynamics illustrated in (17) and (19) is defined and represented by

22 $$ {\mathcal{H} } = {\upsigma }_{1} C_{I} + {\upsigma }_{2} { }H_{C} + {\upsigma }_{3} I_{AC} + {\upsigma }_{4} { }I_{CC} + \frac{{{\mathfrak{B}}_{1} }}{2}{\text{w}}_{1}^{2} + \frac{{{\mathfrak{B}}_{2} }}{2}{\text{w}}_{2}^{2} + \frac{{{\mathfrak{B}}_{3} }}{2}{\text{w}}_{3}^{2} + \frac{{{\mathfrak{B}}_{4} }}{2}{\text{w}}_{4}^{2} + \mathop \sum \limits_{i = 1}^{11} {\Delta }_{i} {\uppsi }_{i} , $$

where ${\uppsi }_{i}$ stands Eq. (19) right hand side $i{\text{th}}$ state variable and ${\Delta }_{1} \left( t \right),{{\Delta}}_{2} \left( t \right),{{\Delta}}_{3} \left( t \right),{{\Delta}}_{4} \left( t \right),{{\Delta}}_{5} \left( t \right),{{\Delta}}_{6} \left( t \right),{{\Delta}}_{7} \left( t \right),{{\Delta}}_{8} \left( t \right),{{\Delta}}_{9} \left( t \right),{{\Delta}}_{10} \left( t \right)$ and ${\Delta }_{11} \left( t \right)$ are the adjoint state variables. Similarly using the same method stated in,^1,4,33,36^ we obtain the co-state variables with the existence condition explained by theorem below:

#### Theorem 10

Let $${\text{w}} = { }\left( {{\text{w}}_{1}^{*} ,{\text{ w}}_{2}^{*} ,{\text{w}}_{3}^{*} ,{\text{ w}}_{4}^{*} } \right)$$ represent the optimal control function and ($$S^{*} ,{ }C_{P}^{*} ,{ }H_{P}^{*} ,C_{V}^{*} ,{ }C_{I}^{*} ,{ }H_{A}^{*} ,{ }H_{C}^{*} ,{ }I_{AC}^{*} ,{ }I_{CC}^{*} ,{ }C_{R}^{*} ,{ }H_{T}^{*} )$$ represent the corresponding unique maximal solutions of the dynamical system (17) at the initial population illustrated in (18) and the objective function stated in (19) at the fixed boundary time $$T_{f}$$ given in (20). Then we need the adjoint functions represented by $${\Delta }_{i}^{*} \left( \cdot \right),{ }i{ } = { }1,{ }...{ },{ }11$$ that corresponds to the equations written by

23 $$ \begin{aligned}   \frac{{d\Delta _{1} }}{{dt}} &  = \left( {1 - w_{1} } \right)\lambda _{H}^{*} \left( {\Delta _{1}  - \Delta _{6} } \right) + \left( {1 - w_{2} } \right)\lambda _{C}^{*} \left( {\Delta _{1}  - \Delta _{5} } \right) + \mu \Delta _{1} , \\    \frac{{d\Delta _{2} }}{{dt}} &  = \left( {1 - w_{1} } \right)\lambda _{H}^{*} \left( {\Delta _{2}  - \Delta _{6} } \right) + \alpha _{1} \left( {\Delta _{2}  - \Delta _{1} } \right) + \mu \Delta _{2} , \\    \frac{{d\Delta _{3} }}{{dt}} &  = \left( {1 - w_{2} } \right)\lambda _{C}^{*} \left( {\Delta _{3}  - \Delta _{5} } \right) + \alpha _{2} \left( {\Delta _{3}  - \Delta _{1} } \right) + \mu \Delta _{3} , \\    \frac{{d\Delta _{4} }}{{dt}} &  = \left( {1 - w_{1} } \right)\lambda _{H}^{*} \left( {\Delta _{4}  - \Delta _{6} } \right) + \left( {1 - \Delta _{2} } \right)\varepsilon \lambda _{C}^{*} \left( {\Delta _{4}  - \Delta _{5} } \right) + \rho \left( {\Delta _{4}  - \Delta _{1} } \right) + \mu \Delta _{4} , \\  \end{aligned}  $$

$$ \begin{aligned}\frac{d{\Delta }_{5}}{dt}& =-{\upsigma }_{1}+\left(1-{{\text{w}}}_{2}\right){\upbeta }_{2}{A}_{1}^{*}\left({\Delta }_{1}-{\Delta }_{5}\right)+\left(1-{{\text{w}}}_{2}\right){\upbeta }_{2}{H}_{{\text{P}}}^{*}\left({\Delta }_{3}-{\Delta }_{5}\right)+\left(1-{{\text{w}}}_{2}\right)\varepsilon {\upbeta }_{2}{C}_{{\text{V}}}^{*}\left({\Delta }_{4}-{\Delta }_{5}\right)\\ & \quad+\left(1-{{\text{w}}}_{2}\right){\phi }_{1}{\upbeta }_{2}{H}_{{\text{A}}}^{*}\left({\Delta }_{6}-{\Delta }_{8}\right)+\left(1-{{\text{w}}}_{2}\right){\phi }_{2}{\upbeta }_{2}{H}_{{\text{C}}}^{*}\left({\Delta }_{7}-{\Delta }_{9}\right)+\left(1-{{\text{w}}}_{1}\right)\upsilon {\lambda }_{H}\left({\Delta }_{5}-{\Delta }_{8}\right)\\ & \quad +\left(\mu +{d}_{1}\right){\Delta }_{5}+{\mathfrak{u}}_{3}\kappa \left({\Delta }_{5}-{\Delta }_{10}\right)\end{aligned} $$

$$ \begin{aligned} \frac{{d{\Delta }_{6} }}{dt} & = \left( {1 - {\text{w}}_{1} } \right)\frac{{\beta_{1} }}{N}S^{*} \left( {{\Delta }_{1} - {\Delta }_{6} } \right) + \left( {1 - {\text{w}}_{1} } \right)\frac{{\beta_{1} }}{N}C_{{\text{P}}}^{*} \left( {{\Delta }_{2} - {\Delta }_{6} } \right) + \left( {1 - {\text{w}}_{1} } \right)\frac{{\beta_{1} }}{N}C_{{\text{V}}}^{*} \left( {{\Delta }_{4} - {\Delta }_{6} } \right) \\ &\quad+ \left( {1 - {\text{w}}_{1} } \right)\upsilon \frac{{\beta_{1} }}{N}C_{{\text{I}}}^{*} \left( {{\Delta }_{5} - {\Delta }_{8} } \right) + \left( {1 - {\text{w}}_{2} } \right)\phi_{1} \lambda_{C}^{*} \left( {{\Delta }_{6} - {\Delta }_{8} } \right) + \left( {\mu + d_{2} } \right){\Delta }_{6} + {\uptheta }\left( {{\Delta }_{6} - {\Delta }_{7} } \right), \end{aligned} $$

$$ \begin{aligned} \frac{{d{\Delta }_{7} }}{dt} & = - {\upsigma }_{2} + \left( {1 - {\text{w}}_{1} } \right)\frac{{\beta_{1} \rho_{1} }}{N}S^{*} \left( {{\Delta }_{1} - {\Delta }_{6} } \right) + \left( {1 - {\text{w}}_{1} } \right)\frac{{\beta_{1} \rho_{1} }}{N}C_{{\text{P}}}^{*} \left( {{\Delta }_{2} - {\Delta }_{6} } \right) + \left( {1 - {\text{w}}_{1} } \right)\frac{{\beta_{1} \rho_{1} }}{N}C_{{\text{V}}}^{*} \left( {{\Delta }_{4} - {\Delta }_{6} } \right) \\ &\quad+ \left( {1 - {\text{w}}_{1} } \right)\upsilon \frac{{\beta_{1} \rho_{1} }}{N}C_{{\text{I}}}^{*} \left( {{\Delta }_{5} - {\Delta }_{8} } \right) + \left( {1 - {\text{w}}_{2} } \right)\phi_{2} \lambda_{C}^{*} \left( {{\Delta }_{7} - {\Delta }_{9} } \right) + \left( {d_{3} + \mu } \right){\Delta }_{7} + {\mathfrak{u}}_{4} \gamma \left( {{\Delta }_{7} - {\Delta }_{11} } \right), \end{aligned} $$

$$ \begin{aligned}\frac{d{\Delta }_{8}}{dt} & = -{\upsigma }_{3}+\left(1-{{\text{w}}}_{1}\right)\frac{{\beta }_{1}{\rho }_{2}}{N}{S}^{*}\left({\Delta }_{1}-{\Delta }_{6}\right)+\left(1-{{\text{w}}}_{2}\right){\upbeta }_{2}{\upomega }_{1}{S}^{*}\left({\Delta }_{1}-{\Delta }_{5}\right)+\left(1-{{\text{w}}}_{1}\right)\frac{{\beta }_{1}{\rho }_{2}}{N}{C}_{{\text{P}}}^{*}\left({\Delta }_{2}-{\Delta }_{6}\right)\\ &\quad+\left(1-{{\text{w}}}_{2}\right){\upbeta }_{2}{\upomega }_{1}{H}_{{\text{P}}}^{*}\left({\Delta }_{3}-{\Delta }_{5}\right)+\left(1-{{\text{w}}}_{1}\right)\frac{{\beta }_{1}{\rho }_{2}}{N}{C}_{{\text{V}}}^{*}\left({\Delta }_{4}-{\Delta }_{6}\right)+\left(1-{{\text{w}}}_{2}\right)\varepsilon {\upbeta }_{2}{\upomega }_{1}{C}_{{\text{V}}}^{*}\left({\Delta }_{4}-{\Delta }_{5}\right)\\ &\quad +\left(1-{{\text{w}}}_{1}\right)\upsilon \frac{{\beta }_{1}{\rho }_{2}}{N}{C}_{{\text{I}}}^{*}\left({\Delta }_{5}-{\Delta }_{8}\right)+{{\text{w}}}_{3}{\uptheta }_{1}\left({\Delta }_{8}-{\Delta }_{6}\right) +\left(1-{{\text{w}}}_{2}\right){\phi }_{1}{\upbeta }_{2}{\upomega }_{1}{H}_{{\text{A}}}^{*}\left({\Delta }_{6}-{\Delta }_{8}\right)\\ &\quad +\left(1-{{\text{w}}}_{2}\right){\phi }_{2}{\upbeta }_{2}{\upomega }_{1}{H}_{{\text{C}}}^{*}\left({\Delta }_{7}-{\Delta }_{9}\right)+\left(\mu +{d}_{4}\right){\Delta }_{8}+\delta \left({\Delta }_{8}-{\Delta }_{9}\right)\end{aligned} $$

$$ \begin{aligned}\frac{d{\Delta }_{9}}{dt} & = -{\upsigma }_{4}+\left(1-{{\text{w}}}_{1}\right)\frac{{\beta }_{1}{\rho }_{3}}{N}{S}^{*}\left({\Delta }_{1}-{\Delta }_{6}\right)+\left(1-{{\text{w}}}_{2}\right){\upbeta }_{2}{\upomega }_{2}{S}^{*}\left({\Delta }_{1}-{\Delta }_{5}\right)+\left(1-{{\text{w}}}_{1}\right)\frac{{\beta }_{1}{\rho }_{3}}{N}{C}_{{\text{P}}}^{*}\left({\Delta }_{2}-{\Delta }_{6}\right)\\ & \quad +\left(1-{{\text{w}}}_{2}\right){\upbeta }_{2}{\upomega }_{2}{H}_{{\text{P}}}^{*}\left({\Delta }_{3}-{\Delta }_{5}\right)+\left(1-{{\text{w}}}_{1}\right)\frac{{\beta }_{1}{\rho }_{3}}{N}{C}_{{\text{V}}}^{*}\left({\Delta }_{4}-{\Delta }_{6}\right)+\left(1-{{\text{w}}}_{2}\right)\varepsilon {\upbeta }_{2}{\upomega }_{2}{C}_{{\text{V}}}^{*}\left({\Delta }_{4}-{\Delta }_{5}\right)\\ & \quad+\left(1-{{\text{w}}}_{1}\right)\upsilon \frac{{\beta }_{1}{\rho }_{3}}{N}{C}_{{\text{I}}}^{*}\left({\Delta }_{5}-{\Delta }_{8}\right)+\left(1-{\Delta }_{2}\right){\phi }_{1}{\upbeta }_{2}{\upomega }_{2}{H}_{{\text{A}}}^{*}\left({\Delta }_{6}-{\Delta }_{8}\right)+{{\text{w}}}_{3}{\uptheta }_{2}\left({\Delta }_{9}-{\Delta }_{7}\right)\\ & \quad+\left(1-{{\text{w}}}_{2}\right){\phi }_{2}{\upbeta }_{2}{\upomega }_{2}{H}_{{\text{C}}}^{*}\left({\Delta }_{7}-{\Delta }_{9}\right)+\left(\mu +{d}_{5}\right){\Delta }_{9}\end{aligned} $$

$$ \frac{{d{\Delta }_{10} }}{dt} = - {{\eta \Delta }}_{1} + \left( {\mu + \eta } \right){\Delta }_{10} , $$

$\frac{{d{\Delta }_{11} }}{dt} = \mu {\Delta }_{11}$, at the transiversality criteria illustrated by

24 $$ {\Delta }_{i}^{*} \left( {T_{f} } \right) = 0,\;i = 1, 2, \ldots ,11. $$

Furthermore, the associated optimal functions $w_{1}^{*} \left( t \right), w_{2}^{*} \left( t \right), w_{3}^{*} \left( t \right),$ and $w_{4}^{*} \left( t \right)$ are illustrated by

25 $$ \begin{aligned}w_{1}^{*} \left( t \right) &  = \max \left\{ {0,~\min \left\{ {\frac{{\lambda _{H}^{*} S^{*} \left( {\Delta _{6}  - \Delta _{1} } \right) + \lambda _{H}^{*} C_{P}^{*} \left( {\Delta _{6}  - \Delta _{2} } \right) + \lambda _{H}^{*} C_{V}^{*} \left( {\Delta _{6}  - \Delta _{4} } \right) + \upsilon \lambda _{H}^{*} C_{I}^{*} \left( {\Delta _{8}  - \Delta _{5} } \right)}}{{{\mathfrak{B}}_{1} }},1} \right\}} \right\}, \\    w_{2}^{*} \left( t \right) &  = \max \left\{ {0,\min \left\{ {\frac{{\lambda _{C}^{*} S^{*} \left( {\Delta _{5}  - \Delta _{1} } \right) + \lambda _{C}^{*} H_{P}^{*} \left( {\Delta _{5}  - \Delta _{3} } \right) + \varepsilon \lambda _{C}^{*} C_{V}^{*} \left( {\Delta _{5}  - \Delta _{4} } \right) + \phi _{1} \lambda _{C}^{*} H_{A}^{*} \left( {\Delta _{8}  - \Delta _{6} } \right) + \phi _{2} \lambda _{C}^{*} H_{P}^{*} \left( {\Delta _{9}  - \Delta _{7} } \right)}}{{{\mathfrak{B}}_{2} }}~,1} \right\}} \right\}, \\    w_{3}^{*} \left( t \right) &  = \max \left\{ {0,\min \left\{ {\frac{{\Theta _{1} I_{{AC}}^{*} \left( {\Delta _{8}  - \Delta _{6} } \right) + \Theta _{2} I_{{CC}}^{*} \left( {\Delta _{9}  - \Delta _{7} } \right) + \kappa C_{I}^{*} \left( {\Delta _{5}  - \Delta _{{10}} } \right)}}{{{\mathfrak{B}}_{3} }}, 1} \right\}} \right\}, \\  \end{aligned}  $$

$${\text{w}}_{4}^{*} \left( t \right) = \max \left\{ {0,min\left\{ {\frac{{\gamma H_{P}^{*} \left( {{\Delta }_{7} - {\Delta }_{11} } \right)}}{{{\mathfrak{B}}_{4} }}{ },1} \right\}} \right\}$$.

## Numerical simulations

In this section, we perform detailed numerical simulations of the model (3) and the control problem (17) to better understand the system dynamics and identify the most effective optimal control measures that affect the HBV and COVID-19 co-epidemic transmission in the community. Numerical simulations provide visual representations, offering an intuitive understanding of how various parameters impact outbreak dynamics and serving as practical tools for scenario assessment. The utilization of the ODE45 solver in MATLAB 2023a for numerical simulations suggests a robust approach to capturing the dynamics of the infectious disease model. ODE45, belonging to the Runge–Kutta family of methods, is recognized for its stability, particularly in handling stiff ordinary differential equations (ODEs). In this section of the study, by collecting parameter values from different published sources we present the numerical simulation results using ODE45 MATLAB programming code and fourth order Runge–Kutta numerical methods. We applied this method since the results of the Runge–Kutta fourth order numerical method give extremely accurate and good outcome. In addition, Runge–Kutta fourth order numerical method requires four evaluations per step and its global truncation error is $O\left( {h^{4} } \right).$

### Numerical simulation of the co-epidemic model (3)

In this part, we use ode45 with fourth order Runge–Kutta numerical approach and applying values of the parameters illustrated in Table 3 we performed simulations to verify the qualitative results and to investigate the impacts of different controlling strategies to tackle the HBV and COVID-19 co-epidemic spreading in the population.

**Table 3:** Values of parameters used for numerical analysis

Parameter	Baseline value of parameter	References
${\Delta }$	500 individuals/day	^54^
$\mu$	(1/76.31)/ day	^26^
$\alpha_{1}$	0.0015/day	Estimated from ^55^
$\alpha_{2}$	0.0004/day	Estimated from ^55^
$d_{1}$	0.0214/day	^26^
$d_{2}$	0.02/day	^26^
$\theta$	0.333/day	^26^
${\uptheta }_{1}$,${\uptheta }_{2}$	(1/3)/day	^26^
$\upsilon$	0.3 dimensionless	^26^
$\phi_{1}$	1 dimensionless	^26^
$\phi_{2}$	1 dimensionless	^26^
$\gamma$	0.5/day	^26^
$d_{2}$	0.02/day	^26^
$d_{3}$	0.0214/day	^26^
$d_{4}$	0.05/day	^26^
$\eta$	0.002/day	Assumed
$\delta$	0.053/day	Assumed
$\varepsilon$	0.002 no unit	Estimated from ^31^
$\beta_{1}$	5.0 × 10^−8^/day	^26^
$\beta_{2}$	6.29 × 10^−8^/day	^26^
$k_{1}$	0.40 no unit	Assumed
$k_{2}$	0.20 no unit	Assumed
$k_{3}$	0.20 no unit	Assumed
$k_{4}$	0.20 no unit	Assumed
$\rho$	0.30 no unit	Assumed
$\kappa$	0.333/day	^26^

**Figure 2:**
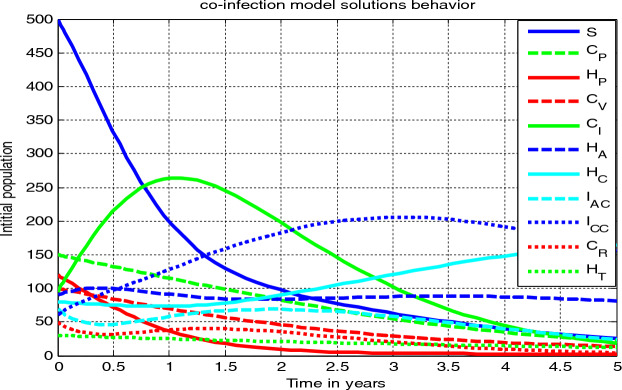
Behaviors of the dynamical system (3) solutions at ${\mathcal{R}}_{{{\text{HC}}}} = 2.68 > 1.$.

Numerical simulation result illustrated by Fig. 2 investigates the behaviours of the complete co-epidemic dynamical system (3) whenever ${ \mathcal{R}}_{{{\text{HC}}}} = {\text{max}}\left\{ {{\mathcal{R}}_{HM} ,{\mathcal{R}}_{CM} } \right\} = \max \left\{ {1.24, 2.68 } \right\} = 3.23 > 1$. The result reveals each of the complete co-epidemic model solution is converging to the complete co-epidemic model endemic equilibrium point. Epidemiologically, one can conclude that the COVID-19 and HBV co-epidemic outbreaks in the population is consistently present but limited to a particular region.

### Simulations of the optimal control problem (17)

In this sub-section, we investigate the effect of intervention strategies on the transmission of HBV and COVID-19 co-epidemic in a population of the study area. The optimal control problem stated in (17)–(21) is solved numerically using ODE45 with the forth order forward and backward Range-Kutta scheme. To verify the qualitative analysis and to investigate the most effective controlling strategy to minimize the number of HBV and COVID-19 co-epidemic people in a population we implemented the numerical simulation of the co-epidemic model using some initial population values and parameter values illustrated in Table 3 and the constants values of $\sigma_{1} = \sigma_{2} = \sigma_{3} = \sigma_{4} = 19$. We implement the numerical simulations in the maximum time level to be five years by considering the following possible illustrated optimal control strategies:

1. Apply single strategy at a time
  (A) Apply HBV protection strategy $\left( {{\text{w}}_{1} \ne 0} \right)$,
  (B) Apply COVID-19 protection strategy ( $w_{2} \ne 0)$,
  (C) Apply HBV treatment strategy ( $w_{4} \ne 0)$,
  (D) Apply COVID-19 treatment strategy ( $w_{3} \ne 0)$.

2. Apply double strategies simultaneously
  (E) Apply protection strategies ($w_{1} \ne 0$ and ${\text{w}}_{2} \ne 0$),
  (F) Apply treatment strategies ($w_{3} \ne 0,$ and ${\text{w}}_{4}$),
  (G) Apply HBV protection and treatment strategies ( $w_{1} \ne 0,$ and ${\text{w}}_{4} \ne 0$),
  (H) Apply COVID-19 protection and treatment strategies ($$w_{2} \ne 0$$ and $$w_{3} )$$,
  (I) Apply HBV protection and COVID-19 treatment strategies ($w_{1} \ne 0$ and $w_{3}$),
  (J) Apply COVID-19 protection and HBV treatment strategies ($$w_{2} \ne 0$$ and $$w_{4} ).$$

3. Triple strategies simultaneously
  (K) Apply HBV and COVID-19 protections and HBV treatment ($w_{1} \ne 0,w_{2} \ne 0,w_{4} \ne 0$).
  (L) Apply HBV and COVID-19 protections and COVID-19 treatment ($w_{1} \ne 0,w_{2} \ne 0,w_{3} \ne 0$).
  (M) Apply HBV protection and COVID-19 and HBV treatments ($w_{1} \ne 0,w_{3} \ne 0, w_{4} \ne 0$).
  (N) Apply COVID-19 protection and COVID-19 and HBV treatments ($w_{2} \ne 0,w_{3} \ne 0,w_{4} \ne 0$).

4. All the possible mentioned strategies simultaneously
  (O) Apply all the four strategies simultaneously ($w_{1} \ne 0,w_{2} \ne 0,w_{3} \ne 0,$ and $w_{4} \ne 0$).

#### Impacts of single strategies on the total co-epidemic population

**Figure 3:**
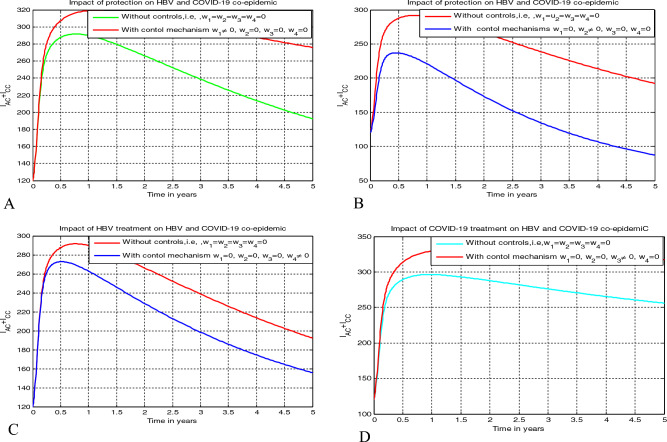
Impact single strategies on the total number of HBV and COVID-19 co-epidemic population.

In this sub-section simulation is done when there is no control strategy in place and considering the following controlling strategies: Strategy A: apply HBV protection, and we present the simulation of optimal control system (17) with $$(w_{1} ) $$ as a protection against HIV infection by Fig. 3A. Strategy B: apply COVID-19 protection strategy, and we present the simulation of optimal control system (17) with protection mechanism ($$w_{2}$$) as a protection against COVID-19 infection by Fig. 3B. Strategy C: apply HBV treatment strategy, and we present the simulation of optimal control system (17) with HBV treatment mechanism ($$w_{4}$$) as a treatment against HBV infection by Fig. 3C. Strategy D: apply COVID-19 treatment strategy, and we present the simulation of optimal control system (17) with COVID-19 treatment mechanism ($$w_{3}$$) as a treatment against COVID-19 infection by Fig. 3D.

From Fig. 3 given presented above we observe that the protective strategies investigated in Fig. 3A and B are more effective strategies as compared to the treatment strategies investigated in Fig. 3C and D. But we recommend that strategy B is the most effective strategy to tackle the co-infection problem in the community.

#### Impacts of double strategies on the total co-epidemic population

**Figure 4:**
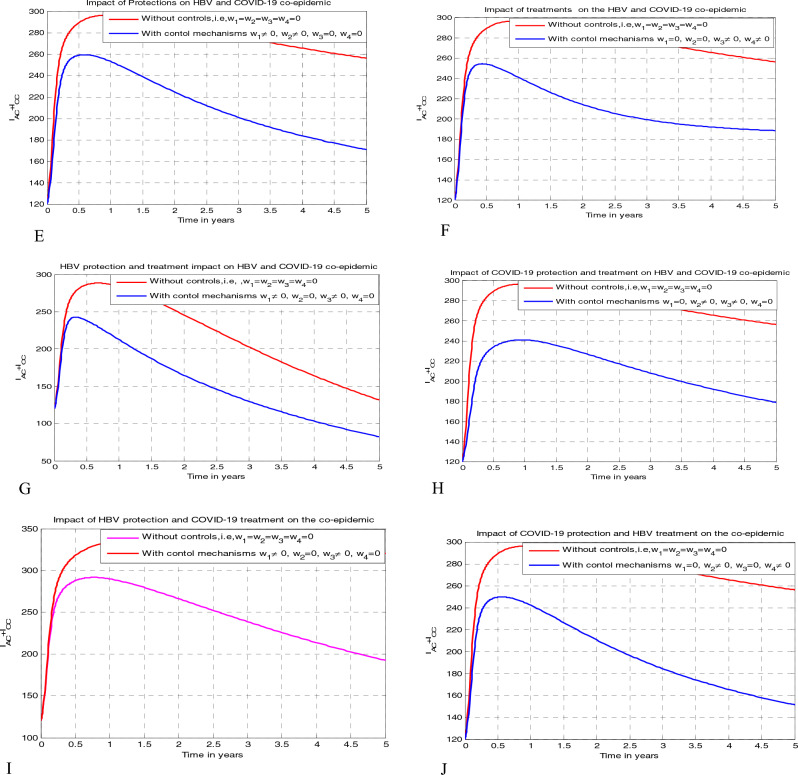
Impacts of double strategies on the number of HBV and COVID-19 co-epidemic population.

In this sub-section simulation is done when there is no control strategy in place and considering the following controlling strategies: Strategy E: apply HBV and COVID-19 protection strategies simultaneously, and we present the simulation of optimal control system (17) with HBV and COVID-19 protection strategies ($$w_{1} $$ and $$ w_{2} )$$ simultaneously as a protection against HBV and COVID-19 infections respectively and is illustrated by Fig. 4E. Strategy F: apply HIV and COVID-19 treatment strategies simultaneously, and we present the simulation of optimal control system (17) with HBV and COVID-19 treatment strategies ($$w_{3} $$ and $$ w_{4} )$$ simultaneously as treatments against COVID-19 and HBV infections respectively and is illustrated by Fig. 4F. Strategy G: apply HIV protection and HBV treatment strategies simultaneously, we present the simulation of optimal control system (17) with HIV protection and HBV treatment strategies ($$w_{1} $$ and $$ w_{4} )$$ simultaneously as a control strategy against HIV and COVID-19 co-infection and is illustrated by Fig. 4G. Strategy H: apply COVID-19 protection and COVID-19 treatment strategies simultaneously, and we present the simulation of optimal control system (17) with COVID-19 protection and COVID-19 treatment strategies ($$ w_{1}$$ and $$ w_{4} )$$ simultaneously as a control strategy against HBV and COVID-19 co-infection and is illustrated by Fig. 4H. Strategy I: apply HBV protection and COVID-19 treatment strategies simultaneously, and we present the simulation of optimal control system (17) with HBV protection and COVID-19 treatment strategies ($$w_{1} $$ and $$ w_{3} )$$ simultaneously as a control strategy against HIV and pneumonia co-infection and is illustrated by Fig. 4I. Strategy J: apply COVID-19 protection and HBV treatment strategies simultaneously, and we present the simulation of optimal control system (17) with COVID-19 protection and HBV treatment strategies ($$ w_{2} $$ and $$ w_{4} )$$ simultaneously as a control strategy against HBV and COVID-19 co-epidemic and is illustrated by Fig. 4J.

From Fig. 4 presented above we observe that the protective and treatment strategies illustrated in Fig. 4G–J are more effective strategies as compared to other strategies investigated in Fig. 4E and F. But we recommend that strategy J is the most effective strategy to tackle the co-infection problem in the community.

#### Impacts of triple strategies on the total number of the co-epidemic

**Figure 5:**
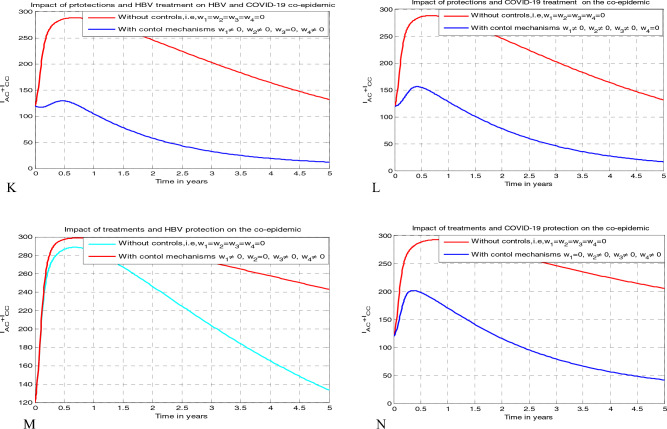
Impacts of triple strategies on the number of HBV and COVID-19 co-epidemic population.

In this sub-section simulation is done when there is no control strategy in place and considering the following controlling strategies: Strategy K: apply both HBV and COVID-19 protections and HBV treatment strategies simultaneously, and we present the simulation of optimal control system (17) with both HBV and COVID-19 protections and HBV treatment strategies ($$w_{1} $$,$$ w_{2}$$ and $$ w_{4} )$$ simultaneously as a control strategy against HBV and COVID-19 co-epidemic and is illustrated by Fig. 5K. Strategy L: use both HBV and COVID-19 protections and COVID-19 treatment strategies simultaneously, and we present the simulation of optimal control system (17) with both HBV and COVID-19 protections and COVID-19 treatment strategies ($$w_{1}$$,$$ w_{2} $$ and $$ w_{3} )$$ simultaneously as a control strategy against HBV and COVID-19 co-epidemic and is illustrated by Fig. 5L. Strategy M: apply both HBV and COVID-19 treatments and HBV protection strategies simultaneously, and we present the simulation of optimal control system (17) with both HBV and COVID-19 treatments and HBV protection strategies ($$ w_{1}$$,$$ w_{3}$$ and $$ w_{4} )$$ simultaneously as a control strategy against HBV and COVID-19 co-epidemic and is investigated in Fig. 5M. Strategy N: apply both HBV and COVID-19 treatments and COVID-19 protection strategies simultaneously, and we present the simulation of optimal control system (17) with both HBV and COVID-19 treatments and COVID-19 protection strategies $$ ( w_{2}$$,$$ w_{3} $$ and $$ w_{4} )$$ simultaneously as a control strategy against HBV and COVID-19 co-infection and is investigated in Fig. 5N.

From Fig. 5 illustrated above we observe that the protections and treatment strategies investigated in Fig. 5K and L are more effective strategies as compared to the strategies investigated in Fig. 5M and N. But we recommend that the strategy investigated in Fig. 5K is the most effective strategy to tackle the HBV and COVID-19 co-epidemic problem in the community.

#### Simulation of the co-infection with strategy O

**Figure 6:**
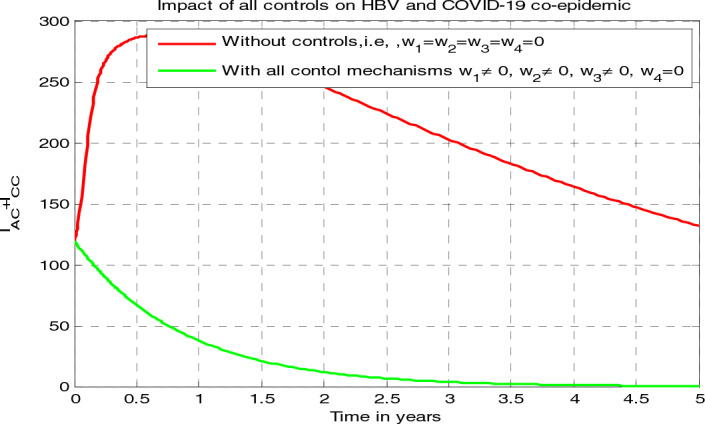
Simulation of total population $(I_{AC} + I_{CC} )$ with all controlling strategies.

In this sub-section numerical simulation is carried out when there is no control strategy in place and when there are controls involving protection and treatment strategies for both COVID-19 and HBV single infections. Figure 6 shows the result that if all the protection and treatment strategies efforts are implemented, the number of individuals co-epidemic with HBV and COVID-19 decreases drastically to zero after 3. Using the result given in Fig. 5 we also compared strategy O to each of other strategies and found out that the strategy shows a significant decline in the number of HBV and COVID-19 co-epidemic individuals and hence strategy O is the most effective strategies to tackle the co-epidemic spreading in the community.

## Conclusion

In this study, we formulated and analyzed the transmission dynamics of HBV and COVID-19 co-epidemic model to achieve our main objective of the study that aims to investigate the impacts of the four time dependent proposed optimal control strategies. The study proved the HBV and COVID-19 co-epidemic model solutions well posedness, stabilities of the equilibrium points and carried out other qualitative and numerical results that contributes to understand the behaviors of real-world disease dynamics, and enhancing the model's reliability and predictive capabilities. Additionally, our qualitative analyses reveal significant insights into the stability of the co-epidemic model. Notably, the HBV, COVID-19, and the HBV and COVID-19 co-epidemic disease-free equilibrium points is demonstrated to be locally asymptotically stable whenever the associated effective reproduction numbers are less than unity, underscoring conditions conducive to disease control. But, the COVID-19 sub-model and the co-epidemic model disease-free equilibrium points reveal the phenomenon of backward bifurcation whenever the corresponding effective reproduction number is less than unity.

The study re-formulated the proposed co-epidemic model optimal control problem by considering four time dependent optimal control strategies and to further enrich our understanding, we conducted comprehensive numerical simulations for the HBV and COVID-19 co-epidemic model, presenting graphical illustrations accompanied by detailed discussions. Our exploration extends to the nuanced behavior of model solutions, emphasizing the impact of optimal control strategies on the HBV and COVID-19 co-epidemic spreading in the community. A noteworthy aspect of this study is the incorporation of the acute and chronic HBV infection stages, incorporating protection for both infections, and vaccination for COVID-19 infection only and these makes our model novel as compared with previously formulated models for HBV and COVID-19 co-infections by other scholars. This novel approach brings a fresh perspective to the investigation of HBV and COVID-19 transmission dynamics in the community. These findings not only contribute to the scientific understanding of HBV and COVID-19 co-epidemic transmission dynamics but also hold significance for scholars engaged in studying the infection spreading within community. The insights gained from this research lay the groundwork for more nuanced and targeted interventions aimed at effectively managing and mitigating HBV and COVID-19 co-epidemic transmission in diverse populations.

The main finding of this study is implementation of vaccination, protections and treatments control measures simultaneously is the best optimal strategy with respect to both the economic and epidemic aspects as compared with other optimal control strategies and hence we recommend for the public health stakeholders to give serious attention regarding maximizing these combined optimal control measures to minimize the HBV and COVID-19 co-epidemic spreading in the community.

Limitation and future work of the proposed study: we haven’t fit data due to the absence of suitable data for model calibration; the focus of the study is on theoretical developments, methodological advancements and exploring the impact of four time dependent optimal control strategies by applying parameter values adopted from published sources rather than on direct applicability to real-world data. The complexity of the HBV and COVID-19 co-epidemic model has been a challenge to find appropriate datasets that capture all relevant variables. For future work, since this study is not exhaustive other potential scholars in the area can modify the proposed HBV and COVID-19 co-epidemic model by incorporating additional aspects such as the stochastic approach, fractional order approach, age structure of individuals, roles of media, roles of the community, HBV vaccination, and fitting the model with appropriate real data.

## Data Availability

Data used to support the findings of this study is incorporated in the article.

## Abbreviations

HBV: Hepatitis B virus
COVID-19: Coronavirus disease of 2019
